# Combinatorial Interactions Are Required for the Efficient Recruitment of Pho Repressive Complex (PhoRC) to Polycomb Response Elements

**DOI:** 10.1371/journal.pgen.1004495

**Published:** 2014-07-10

**Authors:** Tatyana G. Kahn, Per Stenberg, Vincenzo Pirrotta, Yuri B. Schwartz

**Affiliations:** 1 Department of Molecular Biology, Umeå University, Umeå, Sweden; 2 Department of Molecular Biology and Biochemistry, Rutgers University, Piscataway, New Jersey, United States of America; 3 Computational Life Science Cluster (CLiC), Umeå University, Umeå, Sweden; The University of North Carolina at Chapel Hill, United States of America

## Abstract

Polycomb Group (PcG) proteins are epigenetic repressors that control metazoan development and cell differentiation. In *Drosophila*, PcG proteins form five distinct complexes targeted to genes by Polycomb Response Elements (PREs). Of all PcG complexes PhoRC is the only one that contains a sequence-specific DNA binding subunit (PHO or PHOL), which led to a model that places PhoRC at the base of the recruitment hierarchy. Here we demonstrate that *in vivo* PHO is preferred to PHOL as a subunit of PhoRC and that PHO and PHOL associate with PREs and a subset of transcriptionally active promoters. Although the binding to the promoter sites depends on the quality of recognition sequences, the binding to PREs does not. Instead, the efficient recruitment of PhoRC to PREs requires the SFMBT subunit and crosstalk with Polycomb Repressive Complex 1. We find that human YY1 protein, the ortholog of PHO, binds sites at active promoters in the human genome but does not bind most PcG target genes, presumably because the interactions involved in the targeting to *Drosophila* PREs are lost in the mammalian lineage. We conclude that the recruitment of PhoRC to PREs is based on combinatorial interactions and propose that such a recruitment strategy is important to attenuate the binding of PcG proteins when the target genes are transcriptionally active. Our findings allow the appropriate placement of PhoRC in the PcG recruitment hierarchy and provide a rationale to explain why YY1 is unlikely to serve as a general recruiter of mammalian Polycomb complexes despite its reported ability to participate in PcG repression in flies.

## Author Summary

Polycomb Group (PcG) proteins are epigenetic repressors essential for development and cell differentiation. PcG proteins form five complexes targeted to specific genes by Polycomb Response Elements (PREs). How PcG complexes are recruited to PREs is poorly understood. Here we investigate the recruitment of PhoRC, a seemingly simple case of a complex that contains a sequence-specific DNA binding subunit: PHO (or the related protein PHOL). Unexpectedly, we find that the sequence specific binding of PHO is not a primary determinant for recruitment of PhoRC to PRE, which depends on the non-DNA binding subunit SFMBT and cross-talk with another PcG complex, PRC1. The binding of PhoRC is helped by PRC1 and, in turn, may stabilize the binding of PRC1. We propose that the recruitment based on combinatorial interactions enables the conditional binding of PcG proteins, which is important for switching the state of the target genes from repressed to active. The critical role of the cross-talk between PhoRC and PRC1 is further supported by the finding that in mammals, where the protein domains linking the two complexes are missing, the PHO ortholog YY1 has no implication in PcG repression, despite 100% conservation between DNA binding domains of YY1 and PHO.

## Introduction

Polycomb Group (PcG) proteins are critical regulators of metazoan development and cell differentiation [1]–[4]. Their best understood role is to repress key developmental genes that trigger alternative programs of genome expression in cells where these programs should not be executed [4]. Polycomb proteins act as multisubunit complexes of which Polycomb Repressive complexes (PRC) 1 and 2 are the best characterized. PRC2 is a histone methyltransferase specific for lysine 27 of histone H3 (H3K27) [5]–[8] and PRC1 is a histone monoubiquitylase specific for lysine 119 of histone H2A (H2AK119) [9], [10]. PRC1 can recognize the tri-methylated H3K27 (H3K27me3) produced by PRC2 via the chromodomain of its Polycomb subunit. In addition to PRC1 and PRC2, three other complexes, PhoRC, dRAF and PR-DUB have been implicated in PcG regulation in *Drosophila* where PcG repression was first discovered and most studied [11]–[13]. dRAF shares some subunits with PRC1 and is also a H2AK119 monoubiquitylase while PR-DUB possesses a specific H2AK119 de-ubiquitylase activity [12], [13].

The process by which PcG complexes target specific genes is not well understood. In *Drosophila*, regulatory elements termed Polycomb Response Elements (PRE) are the sites where PcG complexes are recruited to repress neighboring genes [14], [15]. Several sequence-specific DNA-binding proteins such as ZESTE, GAGA factor (GAF), PIPSQUEAK, DSP-1, ADF1, PLEIOHOMEOTIC (PHO) and PLEIOHOMEOTIC-LIKE (PHOL) have been proposed to act as PcG recruiters. These proteins often bind at known or presumptive PREs but, with the exception of PHO/PHOL, their genetic ablation does not lead to the de-repression of *HOX* genes (the classical readout of PcG loss of function), suggesting that their individual contribution to the repression is not critical. PhoRC is a heterodimer between PHO and SFMBT proteins [11]. It is naturally expected that the recruitment of PhoRC to PREs is mediated by the sequence-specific binding of PHO to DNA. Supporting this notion, the mutation of cognate DNA-binding motifs impairs PcG repression and PhoRC binding to PRE-containing transgenes [16], [17]. PHO and PHOL are partially redundant but PHO is more important for the repression *in vivo* [18].

Aside from PhoRC, none of the PcG complexes contain DNA-binding proteins as stable components and it is not known how they are recruited. It was proposed that the recruitment of these PcG complexes is mediated by multiple weak interactions with a combination of sequence-specific DNA-binding proteins some of which may still be unknown [14], [15]. An alternative hypothesis puts PHO at the base of the recruitment hierarchy [19]. In this view PHO is capable of direct interaction with PRC2 subunits ESC and E(Z), which leads to the recruitment of PRC2 to PREs. Once at the PREs, PRC2 tri-methylates histone H3 at K27 which, in turn, recruits PRC1 via interaction between H3K27me3 and the chromodomain of PC. Although appealing, this hypothesis is contradicted by much of the experimental data. First, in *Drosophila* the regions enriched in H3K27me3 are broad domains while the binding of PRC1 is localized at PREs, which are themselves depleted of nucleosomes and thus poor in H3K27me3 [20]–[22]. Second, some distinct target genes in *Drosophila* bind PRC1 but lack PRC2 or H3K27me3 [20]. In addition, the direct interaction between PHO and PRC2 was questioned [11] and instead the repressive function of PhoRC was attributed to interactions of the MBT domains of SFMBT with surrounding chromatin [11].

If PhoRC is not at the base of the recruitment hierarchy why is its recruitment different from that of other PcG complexes? Is there a rationale to use a combination of individually weak interactions as opposed to the recruitment via a stably associated DNA binding subunit? To gain insight into these questions we took a closer look at the recruitment of the *Drosophila* PhoRC to chromatin. We show that PHO is favored over PHOL as a subunit of PhoRC and that PHO and PHOL associate with PREs but also with a set of sites in the vicinity of transcriptionally active promoters. Surprisingly, while the binding to the promoter proximal sites is driven by the quality of the PHO/PHOL recognition DNA sequence, PREs have only sub-optimal DNA binding sites. Instead the efficient binding of PhoRC to PREs is critically dependent on the SFMBT subunit and is impaired in cells lacking PRC1. We find that human Yin Yang1 (YY1) protein, the ortholog of PHO, retains the ability to bind recognition sequences in the vicinity of active promoters but does not bind to PcG target genes. We argue that the interactions involved in the targeting to PREs in *Drosophila* are not preserved in the mammalian lineage. We conclude that, similar to that of other PcG complexes, the recruitment of PhoRC to PREs relies on combinatorial interactions and propose that this mode of recruitment is important to attenuate the binding when a target gene is de-repressed.

## Results

To better understand the recruitment of different PhoRC variants to chromatin we mapped the genome-wide distribution of PHO, PHOL and SFMBT in a clonal derivative of *Drosophila* cultured embryonic *Schneider L2* cells (Sg4 cells) using chromatin immunoprecipitation (ChIP) analyzed by hybridization to genomic tiling arrays (ChIP-chip). We have previously mapped several PcG and TrxG proteins, RNA Polymerase II (Pol II) and key histone marks [20], [23] in these cells, making them a model system of choice. Using a homogeneous population of cultured cells, as opposed to whole embryos or imaginal discs, allows direct comparison of binding profiles as Polycomb target genes are in the same transcriptional state in all cells [23].

**Figure 1:**
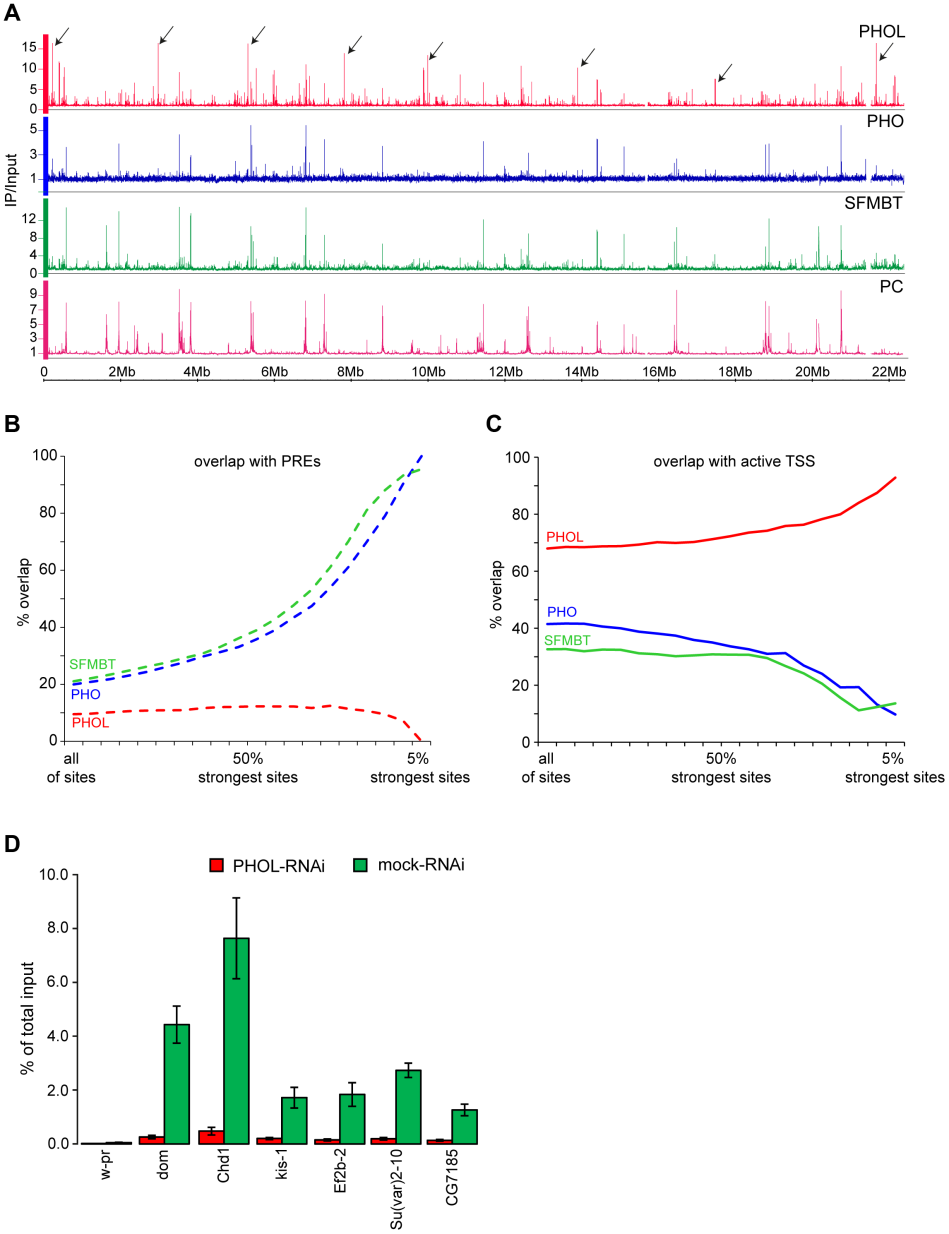
PHO is more tightly linked to SFMBT and PcG repression than PHOL. **A**. The distributions of PHOL (red), PHO (blue) SFMBT (green) and Polycomb (PC, purple) are plotted along the left arm of Chromosome 2. The strongest PHO and SFMBT sites coincide with each other and with PC while the strongest PHOL sites, some of which are marked with black arrows, are outside of PC- regulated genes. **B**. The % overlap of PHOL, PHO and SFMBT binding sites with PREs was plotted as the weaker binding sites were progressively removed from the data sets. **C.** Plotted as in (**B**) the % overlap of PHOL, PHO and SFMBT binding sites with regions +/− 300 bp around active TSSs. Note that the strongest PHO and dSFMBT binding sites are at PREs while the strongest PHOL sites are at active TSS. PREs (total 201) were taken from [23]. The active TSSs (total 7946) were defined based on RNA-sequencing data from Cherbas et al. [32] and a threshold of 300 reads per kb of exon model (RPKM). **D**. The sharp drop in PHOL ChIP signal after PHOL RNAi at a set of TSS-proximal sites (indicated below the x-axis) indicates that they represent a class of genuine PHOL binding sites. The *w-pr* amplicon corresponds to the promoter of the *white* gene and serves as a negative control. The ChIP yields are expressed as fractions of total input material present in the reactions. Here and below the mean of two to three independent experiments and the standard deviation (error bars) are shown.

As expected for components of a protein complex that binds DNA in a sequence-specific fashion, PHO, PHOL and SFMBT associate with discrete sets of narrow binding regions that overlap to a high degree (Figure 1A). 90% of Polycomb target regions, defined as domains of H3K27me3 that overlap with binding sites of PC and E(Z) [20], [23], have at least one detectable binding site co-occupied by PHO, PHOL and SFMBT. This indicates that most of the Polycomb target genes are controlled by at least one PRE that recruits some level of PhoRC. The strength of ChIP-chip signals for PHO, PHOL and SFMBT at individual PREs (here computationally defined as overlapping peaks of E(Z), TRX and PC and coinciding with H3K27me3 domains) varies greatly (∼10 fold). The ChIP-chip signals are poorly correlated with those for PC or E(Z) and, at some PREs, are barely above background (Table S1). This is consistent with previous conclusions that PhoRC is a complex distinct from PRC1 or PRC2 and is not likely to be the principal factor involved in their recruitment, or at least not at all sites [11].

Consistent with the common sequence specificity of PHO and PHOL [18], [24] the relative strength of their ChIP-chip signals at different PREs correlates extremely well (Spearman correlation coefficient = 0.85; Table S2) indicating that PREs do not differ in their preference for PHO *versus* PHOL. It is clear, however, that despite excellent correlation at PREs the overall genomic distributions of PHO and PHOL are very different. The strongest ChIP-chip signals for PHO correspond to PREs and coincide with those for SFMBT while the strongest ChIP-chip signals for PHOL are outside Polycomb target regions and reside within 300 bp of Transcription Start Sites (TSS) of a subset of transcriptionally active loci (Figure 1A–C). We will further refer to these sites as TSS-proximal and note that they also precipitate with antibodies against PHO and SFMBT but only weakly, compared to PREs (Figure 1A–C). The reduction of PHOL precipitation at TSS-proximal sites after RNAi knock-down indicates that these are genuine binding sites and not a product of antibody cross-reactivity (Figure 1D). A similar difference in the genomic binding of PHO and PHOL was previously noted in the mixed population of embryonic cells [25]. We hypothesized that, although both PHO and PHOL are capable of forming a complex with SFMBT when co-expressed in the baculoviral system [11], *in vivo*, PHO is the preferred component of PhoRC. This is consistent with an earlier observation that the interaction of PHO and PHOL with SFMBT is mutually exclusive and that more PHO co-immunoprecipitates with SFMBT from nuclear extracts [11].

### Comparison of TSS-proximal and PRE binding sites

The function of TSS-proximal PHO and PHOL binding sites is unknown. Interestingly, PHO, but apparently not PHOL has also been found to be a component of the INO80 complex [11], which has a nucleosome remodeling activity important for DNA damage repair. The mammalian INO80 complex, which contains the PHO ortholog YY1, has also been reported as an essential co-activator of many genes [26]. Might the TSS-proximal binding of PHO and PHOL be related to this function? The loss of PHOL or/and PHO after corresponding single and double RNAi knock-downs in cultured cells has no detectable effect on the expression of genes closest to TSS-proximal sites (Figure S1). However, it remains possible that the TSS-proximal sites contribute to the regulation of the neighboring genes in a tissue-specific manner not visible in this experiment.

**Figure 2:**
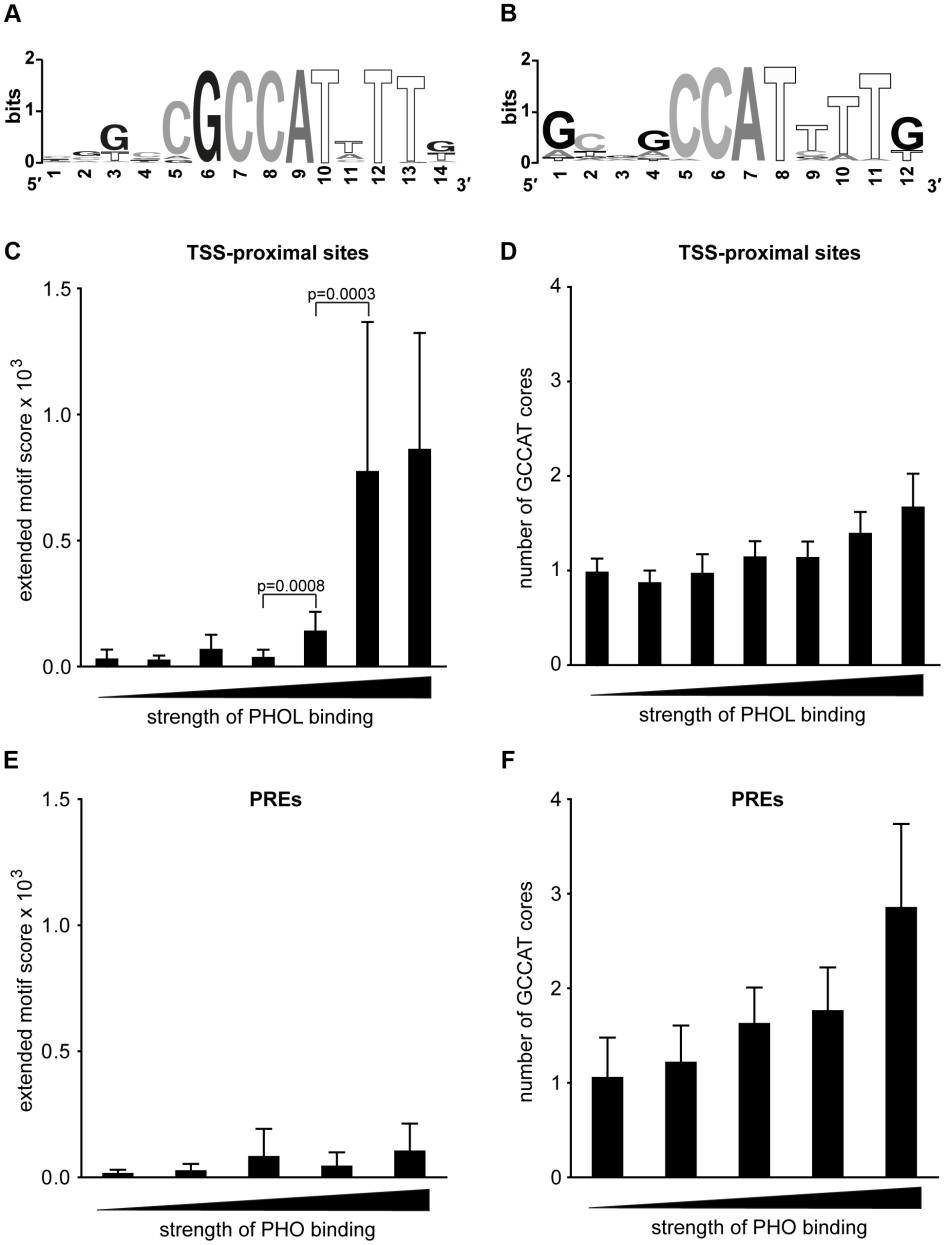
The presence of the extended PHO/PHOL motif does not correlate with efficient PhoRC binding to PREs. **A**. The logo representation of the extended PHO/PHOL motif derived from the analysis of the top 100 strongest TSS-proximal PHOL sites. Note the central conserved GCCAT core between positions 6 and 10. **B**. The logo representation of the sequence motif enriched in the top 100 computationally defined PREs ranked by the strength of PHO ChIP-chip signal. 840 TSS-proximal PHOL binding sites (**C, D**) and 166 computationally defined PREs that show significant binding of PHO in ChIP-chip experiment (**E, F**) were divided in bins according to the strength of PHOL/PHO binding. Histograms show the mean score for the sequences best matching the extended PHO/PHOL motif (**C, E)** or the mean number of conserved GCCAT core sequences (**D, F**) within each bin. Whiskers indicate 95% confidence intervals. There is a clear statistically significant (Wilcoxon rank sum test) trend for the stronger TSS-proximal PHOL binding sites (**C**) to have extended PHO/PHOL motifs. This is in contrast to PREs (**E**) which show no correlation between the strength of PhoRC binding and the presence of extended motifs. We note a weak trend for the stronger binding PREs to have multiple GCCAT cores.

Sequence analysis of the DNA underneath TSS-proximal PHOL sites shows (Figure 2A) that they are enriched in an extended motif (hereafter extended PHO/PHOL motif) that matches the sequence previously defined as a high affinity *in vitro* binding site for YY1, the mammalian ortholog of PHO [27], [24], [28]. The motif has a conserved GCCAT core and a thymidine two nucleotides downstream of the core. A very similar motif was picked up previously by Oktaba *et al.* [29], who analyzed all genomic sites (including both PREs and strong TSS-proximal sites) bound by PHO and SFMBT in *Drosophila* embryonic and larval chromatin. And a similar motif was previously described as characteristic of embryonic PHO binding sites that do not bind PC [30].

The DNA sequences underneath PHO/PHOL-bound PREs are enriched in GA dinucleotide repeats of various lengths, the sequence feature recognized by the GAF protein frequently found at PREs [14], and sequences matching a degenerate version of the extended PHO/PHOL motif (Figure 2B). The latter retains the conserved CAT core but allows variation in all other positions. The 20% of the PREs with the strongest PHO/PHOL ChIP-chip signals are enriched in GCCAT sequences with no nucleotide preference in the flanking positions. Overall the sequence analysis shows no evidence that the recruitment of PhoRC to PREs can be attributed to the extended PHO/PHOL motif or another completely unrelated recognition sequence.

Examination of the ChIP-chip signal strength for PHO or PHOL at TSS-proximal sites reveals a significant correlation with the presence of the extended PHO/PHOL motif and its quality (Figure 2C). Such correlation is expected for a sequence-specific protein whose binding is driven primarily by its interaction with DNA. We note, however, that not all TSS-proximal binding sites, even some of the strongest, contain the extended motif or even a single conserved GCCAT core (Figure 2D). This indicates that in certain instances strong binding is achieved in the absence of both, consistent with earlier reports that, although as a rule the conserved core sequence is critical for high-affinity PHO binding *in vitro*, the substitution of one nucleotide in the first or the second position of the core is tolerated in some cases [16], [31].

In agreement with the sequence motif analysis, the strength of PHO ChIP-chip signals at PREs is not correlated with the presence of the extended PHO/PHOL motif and the majority of the strongest binding PREs lack the extended motif (Figure 2E). Instead, there is a trend for the strongest PHO binding PREs to have multiple conserved GCCAT cores (Figure 2F). We conclude that, while the sequence-specific interactions of PHO/PHOL with DNA appear to be the significant determinant of binding at TSS-proximal sites, some additional interactions are important for binding at PREs.

### SFMBT is required for efficient recruitment of PhoRC to PREs

A drawback of the ChIP approach is that differences in the antibody efficiencies make it impossible to gauge the relative amounts of PHO and PHOL at a given genomic site. Therefore, based on the ChIP data alone, we cannot unequivocally determine whether TSS proximal sites bind the same amount of PHO and PHOL but, comparatively, we can conclude that PREs bind much more PHO than the TSS-proximal sites. We think that equal binding of PHO and PHOL at TSSs is the more parsimonious interpretation. It fits with the similar sequence specificity reported for PHO and PHOL *in vitro* [18] and aligns well with the idea that the binding of both proteins to TSS-proximal sites is driven primarily by their interaction with DNA sequences. The corollary of this interpretation is that the efficient or more stable binding of PHO to PREs requires assistance from other chromatin bound protein(s) or specific chromatin configuration.

**Figure 3:**
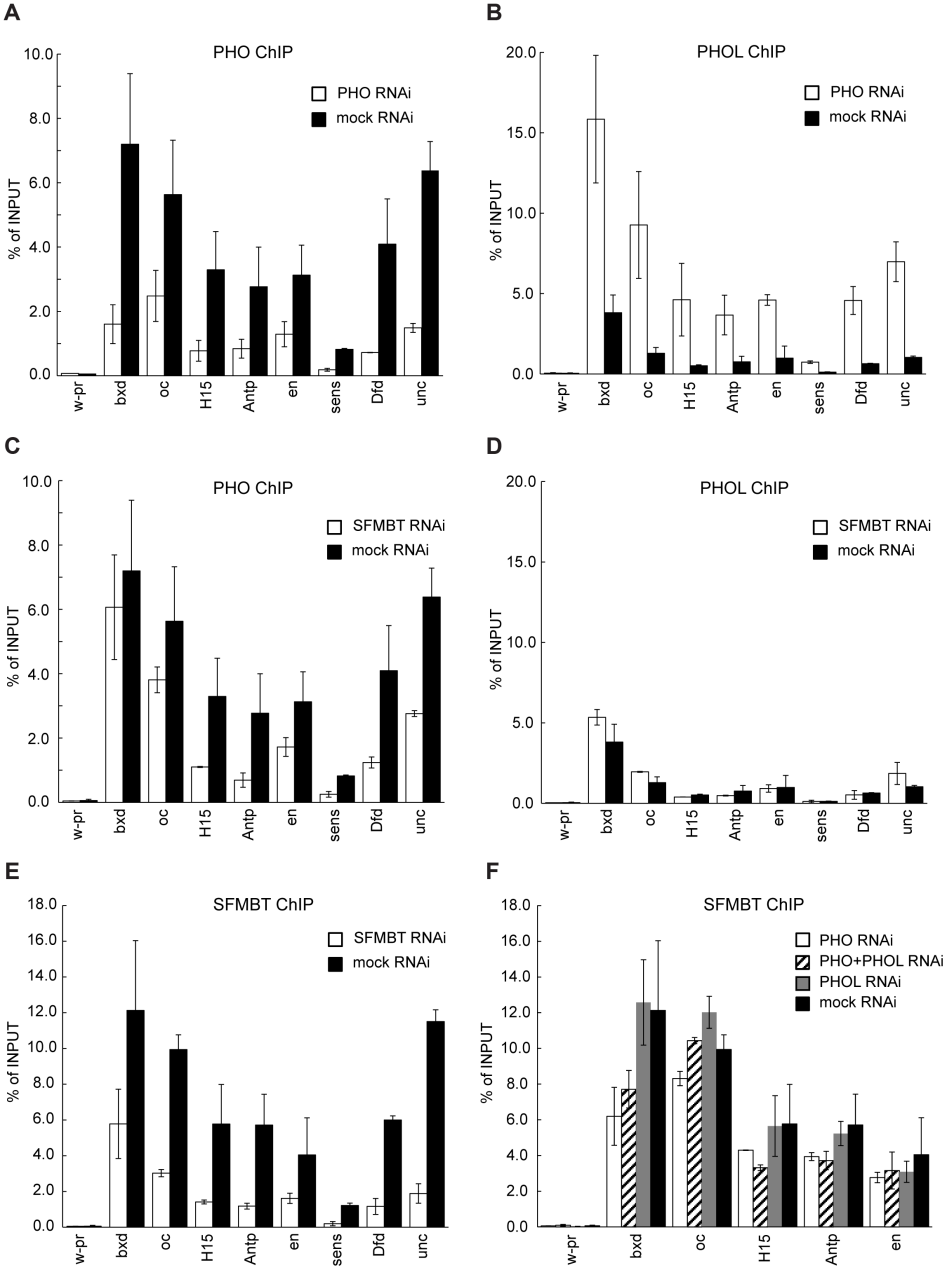
PHO and PHOL compete for interaction with dSFMBT to bind PREs efficiently. Chromatin from cells subjected to RNAi against PHO or dSFMBT was immunoprecipitated with antibodies against PHO (**A**, **C**) or PHOL (**B**, **D**) proteins. The binding of PHO to a selected set of PREs (indicated below x-axes) is reduced after both RNAi treatments (white bars) as compared to mock RNAi (black bars). In contrast, the binding of PHOL increases after PHO (**B**) but not dSFMBT RNAi (**D**). The latter indicates that PHOL competes with PHO for the interaction with dSFMBT to bind PREs more efficiently. The extent of dSFMBT loss from PREs after its RNAi knock-down is shown in (**E**). The effects of PHO (white bars), PHOL (grey bars) or double (shaded bars) RNAi knock-downs on dSFMBT binding are shown in (**F**). In all panels, the “*w-pr*” amplicon, which spans the promoter of the *white* gene, is added as a negative control. For all experiments here and below the efficiency of RNAi knock-downs was gauged by comparing the levels of corresponding proteins in the nuclear extracts from cells treated with mock and specific dsRNAs. The extent of protein depletion in replicate experiments was essentially identical and representative results of western-blot assays are shown on Figures S2 and 4A.

Genetic experiments indicate that PHOL can to a large extent compensate for the loss of PHO [18]. In striking agreement with genetic observations, the knock-down of PHO in ML-DmBG3-c2 cells (hereafter BG3 cells) by RNAi results in dramatic increase of PHOL binding to PREs (Figure 3A, B). The enhanced binding is not paralleled by an increase in the overall PHOL protein level (Figure S2), indicating that PHO normally outcompetes PHOL for binding to PREs. The knock-down of PHO does not enhance the binding of PHOL to TSS-proximal sites (Figure S3) and the reciprocal knock-down of PHOL does not enhance the binding of PHO to either PREs or TSS-proximal sites (Figures S3, S4). This indicates that PHO and PHOL compete specifically for the binding to PREs and suggests that the target of their competition is not the DNA binding sites as such.

**Figure 4:**
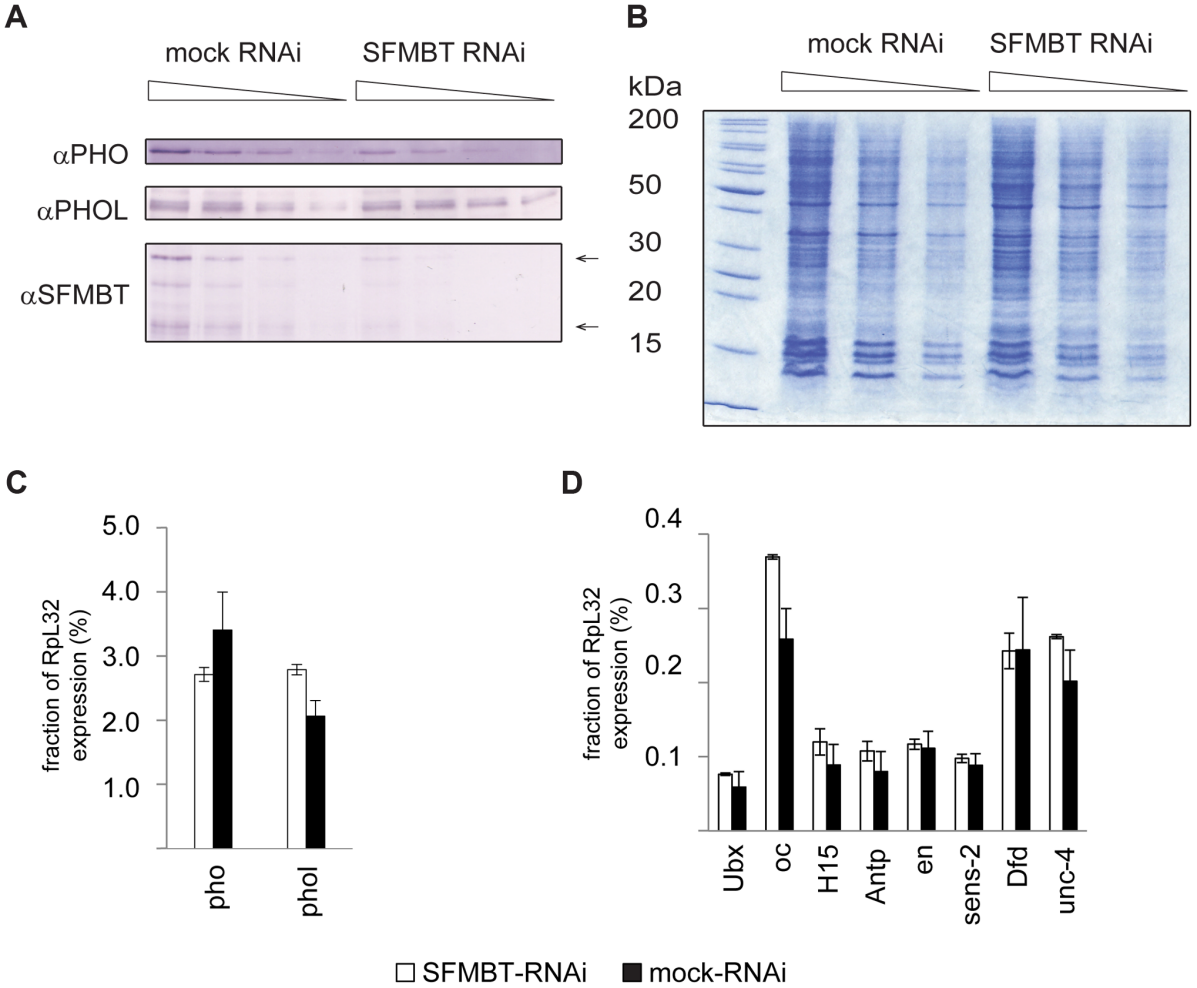
The RNAi knock-down of SFMBT does not affect the abundance of PHO and PHOL. **A.** Serial two-fold dilutions of nuclear protein from mock-treated cells and cells treated with dsRNA against SFMBT were transferred to PVDF membrane and probed with indicated antibodies. Arrows indicate the positions of two SFMBT isoforms. **B**. SDS-PAGE of total nuclear protein followed by coomassie staining was used to control the loading. The weights of molecular standards (in kDa) are shown to the left. As indicated by western blots the RNAi causes roughly four-fold reduction of the SFMBT level, but does not affect the overall abundance of PHOL and causes slight (∼2-fold) reduction of the PHO level. The RT-qPCR analyses indicates that the expression of *pho*, *phol* (**C**) and the set of PcG target genes investigated in this paper (**D**) is also not altered. In **C** and **D** the mean of two independent experiments and the scatter (error bars) are shown.

SFMBT, the other known component of PhoRC, can interact with both PHO and PHOL. We reasoned that SFMBT might be the factor required for their efficient association with PREs. Confirming this conjecture, the knock-down of SFMBT results in reduction of PHO binding to the majority of the PREs tested (Figure 3C, 3E). Importantly, the reduction of PHO binding in this case does not lead to enhanced binding of PHOL (compare Figures 3A and 3C with Figures 3B and 3D), nor is the initial weak PHOL binding to PREs reduced by the knock-down of SFMBT. We speculate that this weak PHOL binding reflects a “basal” level achieved just through interactions with imperfect recognition DNA sequences present at PREs. The effects of SFMBT RNAi cannot be attributed to a reduced expression of the *pho* or *phol* genes or a reduction of the overall levels of PHO and PHOL proteins. Although SFMBT RNAi leads to moderate reduction (∼2-fold) of the PHO level (Figure 4A), the overall abundance of PHOL (Figure 4A) and the expression of *pho* and *phol* genes are unchanged (Figure 4C). Importantly, SFMBT RNAi has no effect on the binding of PHO and PHOL to TSS-proximal sites (Figure S5) indicating that the effect is specific to PREs. We note that in our test system the knock-down of SFMBT did not lead to de-repression of the corresponding target genes (Figure 4D) probably because the required activators are not available in BG3 cells [23]. Therefore the reduced PHO binding and the lack of concomitant increase in PHOL binding to PREs after SFMBT RNAi are not due to the counteraction by transcriptional activity. Overall we conclude that PHO and PHOL compete for the association with SFMBT, which, when available, makes both bind to PREs more efficiently. Given similar expression levels of *pho* and *phol* genes in Sg4 and BG3 cells [32] we suppose that PHO has higher affinity for SFMBT and therefore wins the competition. While the efficient binding of PHO and PHOL to PREs is dependent on SFMBT, we see only marginal reduction of SFMBT binding to PREs after RNAi-mediated knock-down of PHO or even simultaneous knock-down of PHO and PHOL (Figure 3F). The single knock-down of PHOL has no measurable effect on SFMBT binding. These results suggest that factors other than PHO and PHOL contribute to the recruitment of SFMBT to PREs although the full extent of their contribution is difficult to gauge due to the partial nature of RNAi knock-downs.

### Cross-talk between PhoRC and PRC1

**Figure 5:**
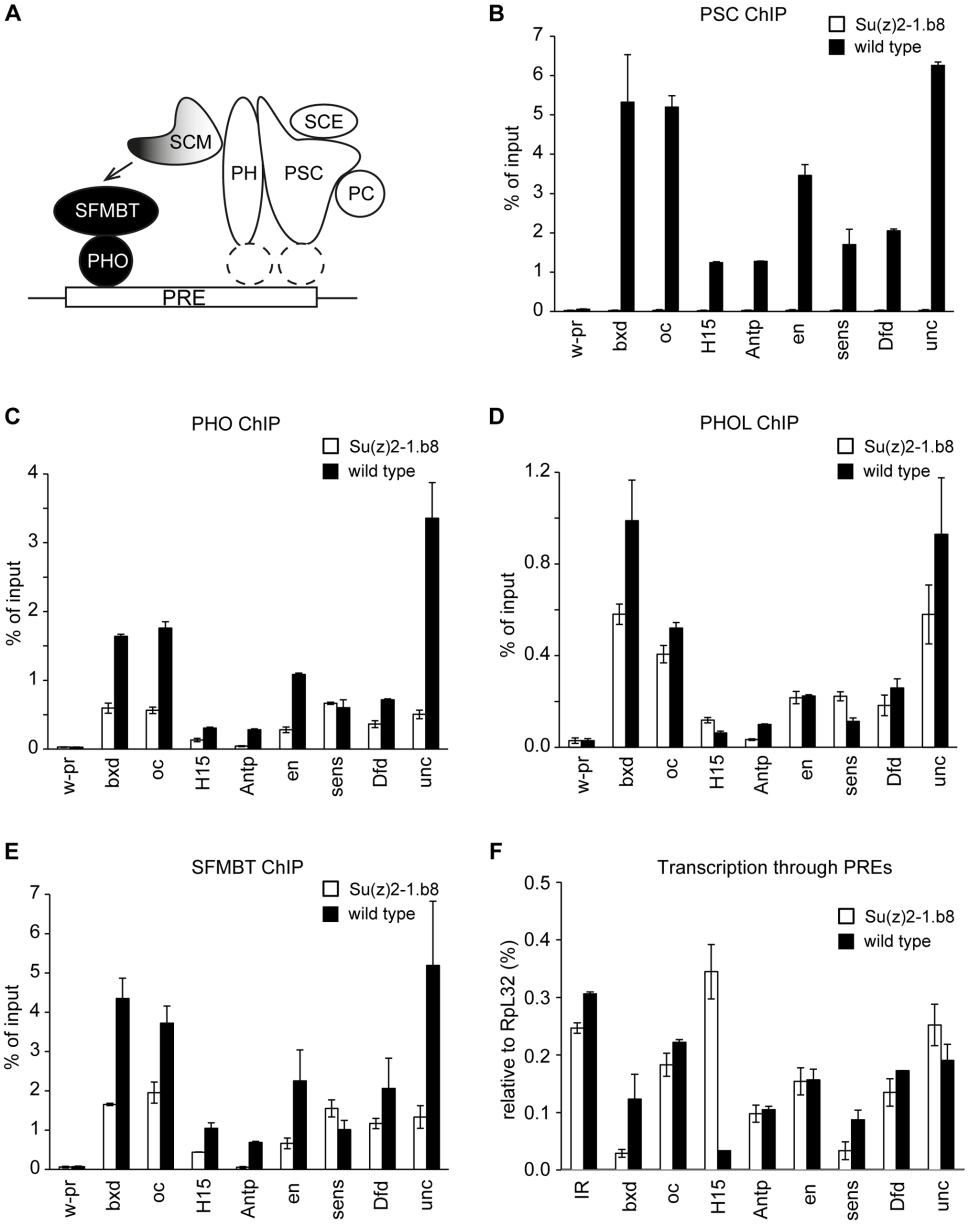
Ablation of PRC1 impairs the binding of PhoRC to PREs. **A.** The model of a cross-talk between PhoRC and PRC1. We propose that relatively weak interactions of PHO with their cognate recognition sequences combine with SFMBT- and SCM-mediated interactions with PRC1 (white shapes represent core components) to result in efficient binding of PhoRC (black circles) to PREs. The recruitment of PRC1 is likely dependent on sequence specific DNA binding proteins, whose identity is currently unknown (dashed white ellipses). Chromatin from cultured cells carrying homozygous *Su(z)2-1.b8* deletion (white bars) or control wild type cells (black bars) was immunoprecipitated with antibodies against PSC (**B**), PHO (**C**), PHOL (**D**) and SFMBT (**E**). Here and in (**F**) the mean result of two independent experiments and the scatter (error bars) are shown. Complete loss of PSC from PREs is paralleled by the reduction of PHO and SFMBT binding from most PREs. The binding of PHOL is not significantly affected. **F.** The reduction of PHO and SFMBT binding in *Su(z)2-1.b8* cells is not paralleled by an enhanced transcription through PREs, which remains low and comparable to that detected at the randomly chosen control intergenic region (IR).

Previous biochemical experiments indicate that SFMBT can directly interact with SCM, a sub-stoichiometric component of PRC1 [33]. This interaction requires N-terminal Zn-finger domains of SFMBT and SCM and is important for PcG repression [33]. We thus envisioned that, at PREs, relatively weak interactions of PHO with its cognate recognition sequences may combine with SFMBT- and SCM-mediated interactions with PRC1 to result in efficient binding (Figure 5A). Such interactions may also contribute to stable binding of PRC1. This model predicts that genetic ablation of PRC1 would impair the binding of PHO and SFMBT at PREs. To test this prediction we used the *Ras* transformation technique [34] to derive cultured cell lines from *Drosophila* embryos homozygous for the *Su(z)2-1.b8* deletion that removes both *Psc* and *Su(z)2* genes [35]. The cell line derived from otherwise wild-type *Ras*-transformed embryos [34] was used as a negative control. In cells lacking PSC/SU(Z)2, the integrity of PRC1 is disrupted (data not shown) and the binding of PSC and other PRC1 components to PREs is abolished (Figure 5B, S6). Consistent with our prediction, the binding of SFMBT and PHO to the majority of the tested PREs is also reduced (Figure 5C, E). The overall effect is statistically significant (p = 0.008, Wilcoxon signed rank test). Notably, the binding of PHOL to the same PREs is not significantly affected (Figure 5D; p = 0.07, Wilcoxon signed rank test). The latter observation reinforces the idea that at PREs the weak binding of PHOL reflects a “basal” level achieved predominantly through interactions with imperfect recognition DNA sequences.

Overall our experiments indicate that the presence of PRC1 stimulates PhoRC binding to most PREs. The available evidence strongly suggests that this is a direct effect. Transcription through PREs, triggered by the loss of PSC/SU(Z)2, could conceivably interfere with PhoRC binding. This, however, is not the case as, in the *Su(z)2-1.b8* cells, we see no increase of the transcriptional activity across a panel of PREs, which remains at the background level seen at the control intergenic region (Figure 5F). The loss of PSC/SU(Z)2 does lead to detectable increase in the transcription of the corresponding PcG target genes but in most cases the transcription remain very low (Figure S6). This and the lack of correlation between the increase in transcriptional activity and the reduction of PhoRC binding to the corresponding PREs argues that the two processes are not mechanistically linked.

### Recruitment via combinatorial interactions may be important for regulated binding of PhoRC to PREs

The binding of PcG proteins is often reduced when the target genes are transcriptionally active and in many instances PRC1 and PRC2 are completely lost from PREs [36], [37], [23]. Our model predicts that in this case the binding of PhoRC to PREs should also be reduced. Such conditional binding would not be possible for PhoRC if the recruitment was constitutively mediated by high-affinity interaction with DNA.

**Figure 6:**
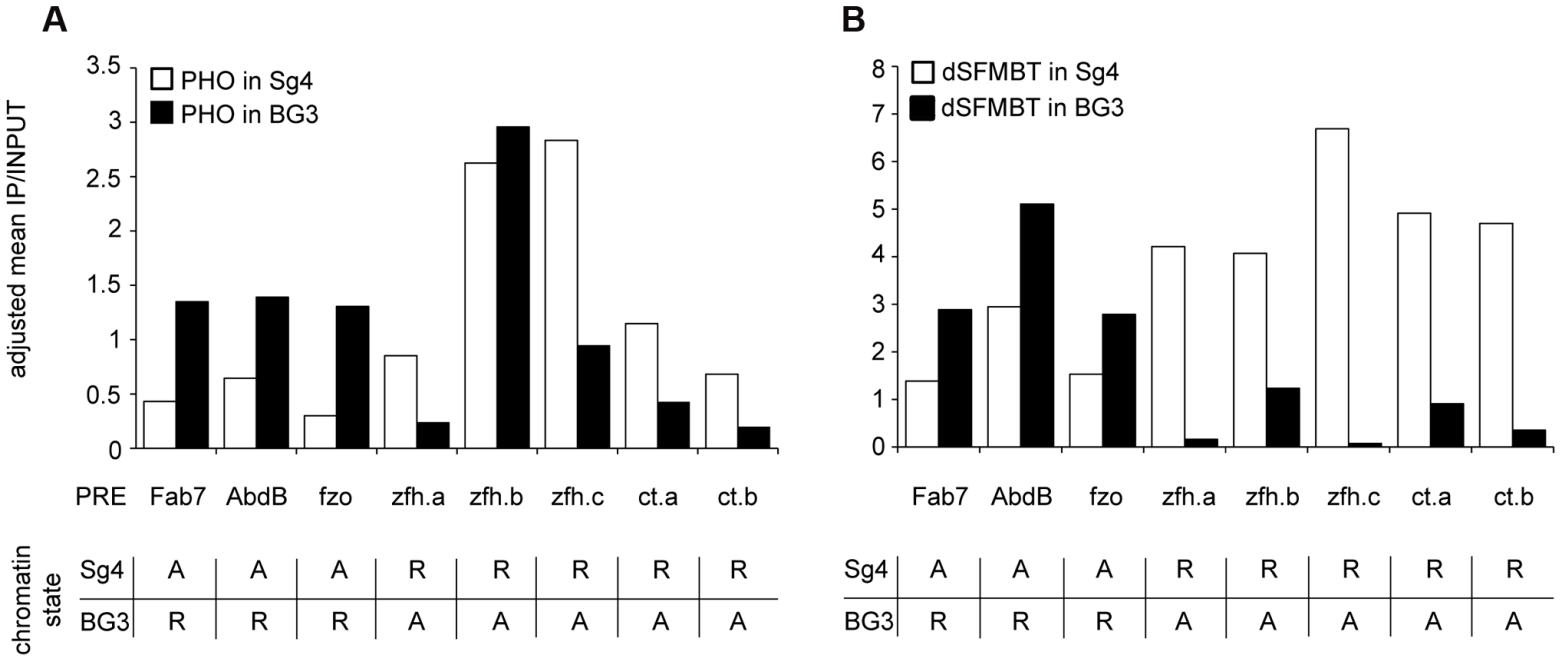
The binding of PHO and dSFMBT to PREs in the active state is significantly reduced. The PHO (**A**) and dSFMBT (**B**) ChIP-chip signals within 500 bp regions centered around PHO peaks at selected PREs (indicated below x-axes) were computed from two independent replicate experiments in Sg4 (white bars) and BG3 cells. The functional state of PREs in each cell line is indicated in the table below each panel (R = repressed; A = active). All PREs display significantly lower dSFMBT binding when in the active state (p = 0.004, Wilcoxon signed rank test). Seven of eight PREs also bind significantly less PHO (p = 0.008, Wilcoxon signed rank test). One PRE (*zfh.b*) shows unusual behavior. When active it binds less dSFMBT but shows no reduction of PHO signal. Inspection of its DNA sequence reveals the presence of three GCCAT cores but no match to the extended PHO/PHOL/YY1 recognition sequence. Conceivably, the advantageous arrangement of multiple GCCAT cores is capable of creating a high-affinity DNA binding platform independent of dSFMBT and of PcG repression.

To investigate this question we took advantage of our previous mapping of PcG target genes residing in alternative chromatin states in Sg4 and BG3 cell lines [23]. We mapped the genomic distribution of PHO in BG3 cells and used the matching SFMBT profile produced with data from the modENCODE Chromatin Consortium [38]. Only about 10% of the Polycomb-repressed genes in one of the two cell lines are active in the other cell line [23] and not all the corresponding PREs bind PhoRC even when repressed. We identified eight PREs that show robust PHO and SFMBT binding in cells where their target genes are repressed and compared their binding properties in cells where the corresponding target genes are active and lost PRC1 and PRC2 (see the list of PREs in Table S3). The comparison of ChIP signals at PREs in two different states shows a clear difference (Figure 6). Unlike PRC1 and PRC2, neither PHO nor SFMBT is completely lost in the active state but their binding is significantly reduced, proportionally more in the case of SFMBT than in the case of PHO. This is consistent with our expectations that when PRC1 and PRC2 are lost, so are the interactions that stabilize SFMBT, leaving only the sequence-dependent binding component. We speculate that the reduction of PhoRC binding is part of the de-repression process and that a recruitment strategy based on combinatorial interactions rather than constitutive binding is essential for the attenuation of the PcG binding.

### YY1 is not implicated in most Polycomb binding sites in human cells

Our observations in *Drosophila* argue that PHO or, in its absence, PHOL has to complex with SFMBT in order to efficiently bind PcG target genes and participate in repression. They also suggest that the efficient binding of PhoRC to PREs depends on the ability of SFMBT and SCM to mediate interactions between PhoRC and PRC1.

YY1 is the mammalian ortholog of PHO [39] that has been traditionally considered a candidate for a sequence-specific DNA-binding recruiter of mammalian PcG complexes. Several lines of indirect evidence support this hypothesis of which by far the strongest is the reported ability of a *YY1* transgene to partially rescue the loss of *pho* function in *Drosophila* [40]. Against this hypothesis are the results of genome-wide mapping in mouse embryonic stem cells, which failed to detect any overlap between YY1 binding sites and PcG target genes [41], [42], and the notion that the SFMBT-SCM link appears to be “lost” in the mammalian lineage. Thus both human orthologs of SCM (SCMH1 and SCML2) lack the N-terminal zinc-finger domain (Figure S7) required for SCM-SFMBT interaction in *Drosophila* [33] and none of the four likely human orthologs of SFMBT have the full assortment of domains present in the *Drosophila* counterpart (Figure S7).

**Figure 7:**
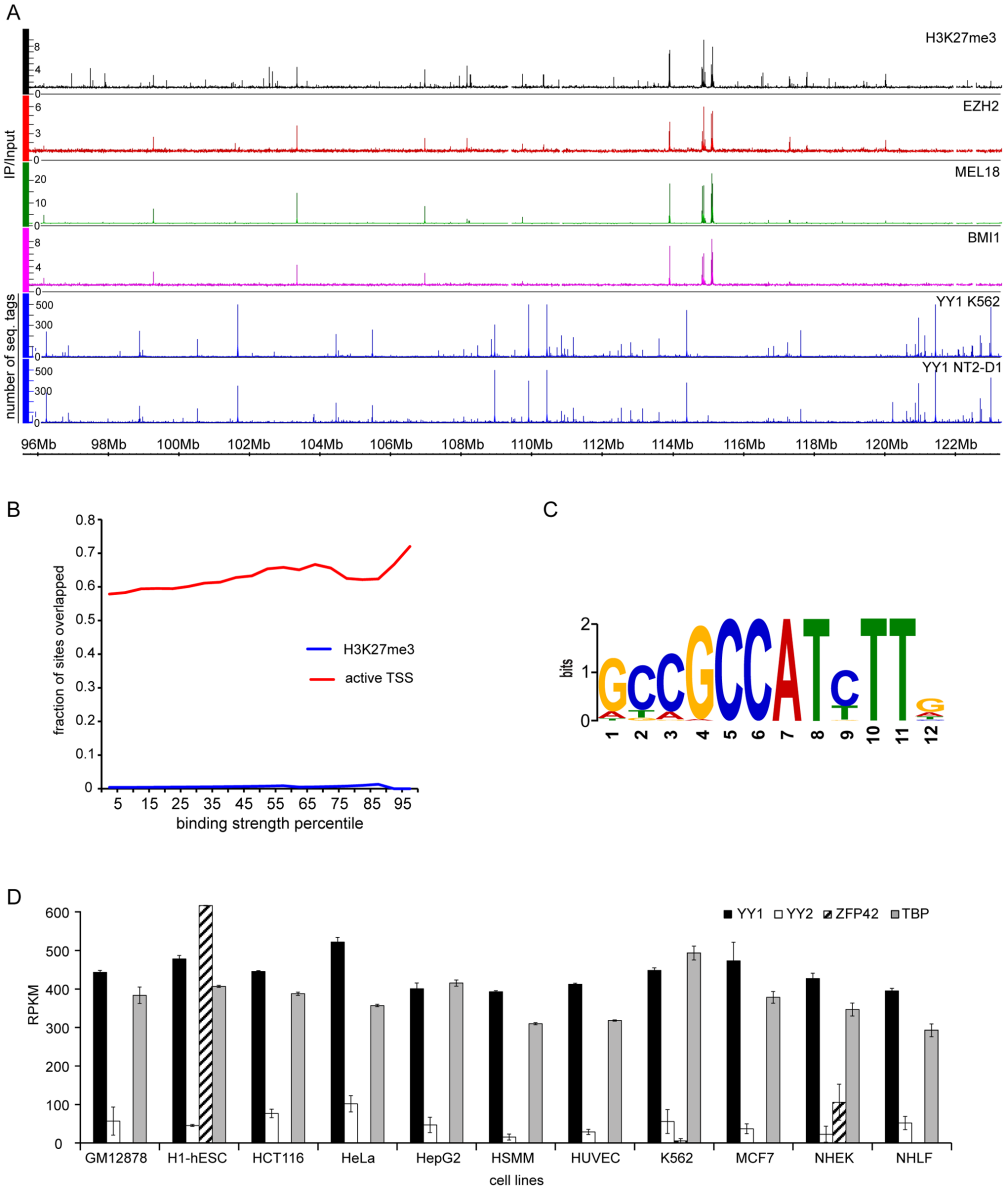
YY1 is not involved in Polycomb silencing in human cells. **A.** The distributions of H3K27me3 (black), EZH2 (red), MEL18 (green) BMI1 (purple) in NT2-D1 cells and the distributions of YY1 in NT2-D1 and K562 cells (blue) are plotted along the 26 Mb segment of Chromosome 12. The strong binding sites for EZH2, MEL18, BMI1 and H3K27me3 coincide and show no overlap with YY1. Note that the distribution of YY1 in NT2-D1 and K562 cells is essentially identical. **B**. The extents of YY1 overlap with H3K27me3 enriched regions (blue line) or regions +/− 700 bp around active TSS (solid lines) in NT2-D1 cells are plotted as function of YY1 binding signal. There is virtually no overlap between YY1 and H3K27me3. The overlap-plots for MEL18, BMI1 and EZH2 are not shown since these have zero values for every bin. The active TSSs were defined based on RNA-sequencing data from ENCODE and the threshold of 300 RPKM. **C.** The logo representation of a sequence motif revealed by the analysis of the DNA underneath YY1 binding sites detected by all three antibodies in NT2-D1 cells. **D**. The expression of *YY1* (black bars), *YY2* (white bars), *Zfp42* (shaded bars) and “housekeeping” *TBP* (grey bars) genes derived from RNA-seq data indicate that YY2 and ZFP42 proteins are not available in most of the cell types listed below the x-axis. The average RPKM values between two independent experiments are plotted with error bars indicating the scatter.

To get additional insight and further test our model we asked whether we could detect the binding of YY1 to PcG target genes in human cells. To this end we mapped the distributions of BMI1, MEL18 (components of human PRC1), EZH2, H3K27me3 (catalytic subunit of human PRC2 and the histone mark it produces) on chromosomes 8, 11 and 12 of human pluripotent embryonic teratocarcinoma NT2-D1 cells using ChIP and hybridization to Affymetrix genomic tiling arrays (ChIP-chip). We then compared the PcG binding profiles to the distribution of the YY1 binding sites in the same cells defined from three independent mapping experiments. The first set of YY1 binding sites was derived from the ChIP and high-throughput sequencing (ChIP-seq) mapping by the ENCODE project [43]. The other two sets were derived from our mapping of YY1 binding to chromosomes 8, 11 and 12 using ChIP-chip with two independently derived antibodies. The latter antibodies are different from the one used by ENCODE. The comparison shows no evidence of YY1 binding to PcG target genes (Figure 7A, B, S8). The result is robust as we see no overlap comparing either the sites common for BMI1, MEL18, EZH2 and H3K27me3 (high-confidence PcG targets) or the distribution of the individual PcG proteins or H3K27me3 mark with any of the individual YY1 profiles or with the set of sites detected by all three antibodies (Figure 7B, S8). Instead we see a strong bias for YY1 to bind a subset of transcriptionally active TSSs (Figure 7B, S8), which show enrichment for the motif [26], [24] previously reported as the optimal YY1 binding site *in vitro* (Figure 7C). We conclude that in NT2-D1 cells YY1 is not bound to genes repressed by PcG and its behavior resembles the sequence-specific binding of *Drosophila* PHO/PHOL to TSS-proximal sites.

NT2-D1 cells are pluripotent [44] and, like mouse embryonic stem cells, may represent a special case where PcG recruitment is independent of YY1. However, examination of YY1 binding in K562 cells, derived from a more differentiated mesoderm lineage, shows a very similar binding profile with the same bias towards transcriptionally active TSSs and no overlap with PcG target genes (Figure 7A, S9). There are three PHO orthologs in human cells: YY1, YY2 and ZFP42 (a.k.a. REX1). If YY1 is not widely involved in PcG repression could YY2 and ZFP42 be implicated in the process instead? Although we cannot fully exclude this (ChIP-grade antibodies against human YY2 and ZFP42 are not available at this time) we think this is very unlikely. Mining the ENCODE RNA expression (RNA-seq) data from eleven different human cell lines indicates that, in contrast to YY1 or PHO, which are ubiquitously expressed, the expression of *ZFP42* gene is restricted to pluripotent embryonic stem cells and the expression of *YY2* is generally low and at the edge of detection in many cell types (Figure 7D).

We conclude that, consistent with the absence of the link between PhoRC and PRC1 and in line with previous reports in mouse embryonic stem cells, the wide implication of YY1-like proteins in PcG repression seen in *Drosophila* is most likely not conserved in humans.

## Discussion

Contrary to initial expectations we found that the recruitment of PhoRC to most PREs is based on combinatorial interactions and not just on the sequence specific binding of PHO to DNA. Thus we see that PREs generally lack the extended PHO/PHOL motif, lose PHO binding after SFMBT knock-down and show reduced PhoRC binding in the absence of PRC1. Overall, the recruitment of PhoRC follows the rules proposed for other PcG complexes, which suggests that it is not likely to be at the base of the recruitment hierarchy. We speculate that in general a recruitment strategy based on combinatorial interactions is required to enable the regulated binding of PcG proteins, which, in turn, is important for switching the state of the target genes from repressed to highly active. This is particularly true if the PcG proteins themselves act as epigenetic marks as was recently suggested [45].

Another important finding of our study is that SFMBT is critical for the efficient binding of PhoRC to PREs and that the recruitment of SFMBT to PREs is only partially dependent on PHO/PHOL. While this paper was in preparation Alfieri and colleagues [46] reported the structural determinants of the interaction between PHO and SFMBT. Consistent with our results they found that a truncation of the SFMBT protein that severely impairs its interaction with PHO leads to only mild reduction of the SFMBT binding to PREs. (see Figure 3B in reference 46). The loss of SFMBT is said to disrupt PcG repression of *HOX* genes to the same extent as simultaneous knock-out of PHO and PHOL [11]. These findings were originally interpreted to indicate that PHO and PHOL serve as sole recruiters of SFMBT, which acts as an effector silencing protein [11]. Although a role of SFMBT as a transcriptional repressor is not excluded, our observations indicate that when SFMBT is lost neither PHO nor PHOL can bind PREs efficiently, which suggests an alternative explanation of the comparable de-repression phenotypes.

We propose that the previously documented interaction between SFMBT and SCM [33] serves as a link between PhoRC and PRC1 and that this link is, at least in part, responsible for the PHO-independent anchoring of SFMBT to PREs. Supporting this idea, genetic ablation of PRC1 leads to significant reduction of SFMBT and PHO binding to most PREs. Consistently, in mammals, where the SFMBT-SCM link is likely broken due to the evolutionary loss of critical N-terminal Zn-finger domain of SCM, YY1 has no wide implication in PcG repression, despite 100% conservation between the DNA binding domains of PHO and YY1.

Whether *in vivo* YY1 forms a complex with either of the four potential mammalian orthologs of SFMBT remains an open question. In *Drosophila* the interaction between PHO and SFMBT is mediated by the evolutionarily highly conserved spacer domain of PHO and the array of four MBT domains of SFMBT [46]. *In vitro*, the corresponding domain of YY1 can interact with MBT domains of L3MBTL2, MBTD1 and SFMBT2 (but not SFMBT1). However, this interaction is ∼50-fold weaker than that of the *Drosophila* counterparts [46]. According to Alfieri et al. [46], the reduced interaction between YY1 and SFMBT orthologs is due to the substitution of critical amino acids within the MBT domains of the latter. It seems, therefore, that YY1 should be able to efficiently interact with *Drosophila* SFMBT. This may explain why YY1, which appears to have no implication in mammalian PcG repression, reportedly rescues homeotic phenotypes of *pho* mutant flies [40]. We hypothesize that YY1 retains the features required to participate in PcG repression provided that the appropriate SFMBT protein and the SFMBT-SCM link are available.

In conclusion, we have significantly advanced our understanding of PhoRC recruitment to PREs and the context within which it contributes to PcG repression. Yet our findings and the study of Alfieri et al. [46] underscore that we still do not understand how PhoRC contributes to the repression. One possibility is that while the binding of PhoRC is helped by PRC1 it, in turn, helps to stabilize the binding of PRC1. This may combine with proposed independent stabilization of PRC1 binding by SCM [47]. It is also unclear whether the kind of contribution provided by PhoRC is dispensable for PcG repression in mammals or whether it is taken over by some other protein complexes.

PHO and PHOL are the only known sequence-specific DNA-binding proteins whose ablation causes the de-repression of *HOX* genes (the classical readout of PcG loss of function) however other DNA-binding proteins like ZESTE, GAF, PIPSQUEAK, DSP-1 and ADF1 are known to contribute to PcG recruitment [14], [15], [48]. The extent of their individual contribution is difficult to gauge because they are involved in additional processes, which, when disrupted, may mask the homeotic phenotypes of the mutant flies. It was proposed that multiple individually weak interactions with a combination of sequence-specific DNA binding proteins mediate the recruitment of PcG complexes [14], [15], [48]. Drawing parallels with PHO and PHOL we should consider the possibility that other sequence-specific DNA binding proteins also bind PREs in combinatorial fashion.

## Methods

### Antibodies

Rabbit polyclonal antibodies against the PHO peptide (amino acids 93–276) and PHOL peptide (amino acids 1–196) were described in [49], [19]. The rabbit polyclonal antibodies against *Drosophila* SFMBT peptide (amino acids 14–113; SFMBT Q2642) were generated as part of the modENCODE project [38] by genetic immunization and affinity purified. Antibodies against EZH2 were from Lake Placid Biologicals (#AR-0163), antibodies against H3K27me3 and MEL18 were purchased from AbCam (#ab6002; # ab16651) and anti-BMI1 antibodies were from Millipore (#05-673). Two independently derived antibodies against YY1 (mouse monoclonal (H-10; #sc-7341) and rabbit polyclonal (C-20; #sc-281) were purchased from Santa Cruz Biotechnology. Their specificity was confirmed by Western blotting with nuclear protein extracts from HEK293T treated with shRNAs against YY1.

### Cell culture and RNAi

The culturing of *Schneider L2* Sg4 clone (also known as SF4) and ML-DmBG3-c2 cells and the dsRNA treatments were done as described in [23]. The derivation of *Drosophila* cultured cell lines homozygous for *Su(z)2-1.b8* deletion was done according to Simcox et al. [34]. Briefly the chromosome carrying *Su(z)2-1.b8* deletion was recombined with a transgene encoding RAS^V12^ (an activated form of RAS locked in the GTP-bound state) driven by *UAS* promoter or with a transgene expressing high level of GAL4 from constitutively active *Act5C* promoter. The fly stocks baring recombinant chromosomes were further crossed to each other to yield embryos that carry homozygous *Su(z)2-1.b8* deletion and a combination of *UAS-RasV12* and *Act5C-GAL4* transgenes, from which the mutant cell lines were derived. NTERA-2 cl.D1 embryonic carcinoma cells (ATCC #CRL-1973, lot#4742175) were cultured in Dulbecco's Modified Eagle's Medium (DMEM, ATCC #30-2002) supplemented with 10% of FBS (ATCC #30-2020) at 37°C in 5% CO_2_ atmosphere.

### Chromatin immunoprecipitation and genome-wide analyses

ChIP, qPCR analysis and hybridization to *Drosophila* tiling arrays v1.0 (Forward) (Cat# 900587; Affymetrix) were done as described [21], [20]. The primers used for qPCR are listed in Supplementary Table S4.

NTERA-2 cl.D1 cells were crosslinked by adding 37% formaldehyde (Sigma) to the final concentration of 1% directly to the cell culture. The crosslinking reaction was performed for 10 min at +37°C on a rocking platform. Cells were permeabilized with 1% SDS, and sheared in TE-PMSF (0.1% SDS, 10 mM Tris-HCl pH 8.0, 1 mM EDTA pH 8.0, 1 mM PMSF) using Bioruptor sonicator (Diagenode) set to the “high power” output. Five 10 minute long sonication sessions of 0.5 minute sonication pulses alternated with 0.5 minute pauses were performed at +4°C. After sonication the cell lysates were brought to RIPA (0.1% SDS, 1% Triton ×100, 0.1% DOC, 10 mM Tris-HCl pH 8.0, 1 mM EDTA pH 8.0, 140 mM NaCl) and spun to remove insoluble debris. ChIP was done as described in Schwartz et al. [20]. To prepare the labeled probe for hybridization to tiling microarrays one third of DNA immunoprecipitated in a ChIP reaction was subjected to whole genome amplification using WGA2 kit (Sigma). Amplification was done according to the manufacturer's instructions with chemical fragmentation step omitted. 8 µg of amplified DNA was fragmented with DNaseI to the average size of 50–100 bp and end-labeled with bio-11-ddATP (Perkin Elmer Cat# NEL548) in TdT (Roche; Cat# 03333566001) catalyzed reaction. Labeled DNA was hybridized to GeneChip Human Tiling 2.0R F arrays (Cat# 900784; Affymetrix) for 18 hours at 45°C with 45 rpm rotation in a mixture containing 1×MES, 3M TMAC (Sigma, Cat# T3411), 40 pM control oligo B2 (Affymetrix Cat# 900301), 50 µg/ml Human Cot-1 DNA (Invitrogen Cat# 15279-011), 50 µg/ml sonicated herring sperm DNA and 0.02% Triton ×100. Fluidics station and EukGE-WS2v4 protocol (Affymetrix) were used for microarray washing and staining.

The primary ChIP-chip data for SFMBT distribution in ML-DmBG3-c2 cells were downloaded from modMINE (http://intermine.modencode.org). The genomic binding profile for a given protein was derived from microarray hybridizations of the DNA from two independent ChIP experiments and two matching chromatin inputs. The distribution profiles were computed as average ChIP to average Input intensity ratios smoothed by taking a trimmed mean over a sliding 675 bp window. Windows with less than 10 features were excluded from the analysis. The results were visualized with the Integrated Genome Browser [50]. For all downstream analyses *D. melanogaster* dm3 (2006) genome assembly and FlyBase v5.5 gene annotation or *H. sapiens* GRCh37/hg19 (2009) genome assembly and UCSC June 2010 gene annotations were used.

### Computational analyses

#### Definition of bound regions

To define the genomic set of bound regions for a given protein first the mean and the standard deviation (SD) of all ChIP/Input hybridization ratios in the corresponding microarray profile was computed and the value of the mean + 3*SD subtracted from each ChIP/Input value to produce adjusted microarray data set. The adjusted data set was further refined by setting all negative values to zero. Then the bound regions were defined as coordinates of clusters of microarray features which satisfied the following two criteria. i) the adjusted ChIP/Input ratios of the features had to exceed 0, ii) the maximum distance to the nearest neighboring feature with an intensity of greater then 0, should not exceed 500 bp. The clusters shorter than 360 bp (estimated ChIP resolution) were excluded from further analysis. For each bound region the cluster of 6 consecutive microarray features with the highest average ChIP/Input hybridization signal was defined to represent the binding peaks. These average ChIP/Input hybridization values were used as a measure of binding strength in overlap comparison tests in Figures 1B, 1C 7B, S8, S9. The bound regions for YY1 in NT2 and K563 cells generated by ENCODE project were downloaded from http://genome.ucsc.edu/ENCODE/.

#### Gene expression analysis

The definition of genes expressed in Sg4 cells was taken from [32]. 75% of all PHOL sites outside of Polycomb target regions reside within 300 bp of an annotated TSS therefore 600 bp regions centered on TSS were used for overlap comparison between transcriptionally active TSS and PHOL, PHO or SFMBT bound regions. The expression status of genes in NTERA-2 cl.D1 cells was determined from expression microarray study by Fong et al. [51]. Genes with an RMA value [52] of more than 6 were defined as expressed. 75% of all YY1 sites were found within 700 bp of an annotated TSS. Therefore 1400 bp regions centered on TSS were used for overlap comparison between transcriptionally active TSS and YY1 bound regions. The RNA-seq data used for analysis in Figure 7D was downloaded from http://genome.ucsc.edu/ENCODE/ and expressed genes defined as described by Djebali et al. [53].

#### Motif definition and scoring

The discovery of enriched sequence motifs was performed using the MEME Suite at http://meme.sdsc.edu [54] separately for top 100 strongest TSS-proximal PHOL sites, top 100 strongest PHO/PHOL sites overlapping computationally defined PREs and high-confidence YY1 sites in NT2 cells. Sequences +/−200 bp around binding peaks were taken as input for the search of motifs between 5 and 15 nucleotides in length accounting for complementary sequence. The eight top scoring motifs were recorded in each search following by removal of low complexity motifs consisting of mono- and di-nucleotide repeats. In each case the removal of low complexity sequences yielded a single motif. Sequence logos were constructed using the WebLogo package [55].

To score for extended PHO/PHOL motifs DNA sequences of selected binding sites were scanned with a 14 bp window slid one nucleotide position at a time. Each window was assigned a score according to the Position Weight Matrix for the extended PHO/PHOL motif (shown on Figure 2A) accounting for complimentary sequence. For each binding site the single best score defined the score for this site. All perfect matches to GCCAT sequence within selected regions were counted to estimate the number of conserved GCCAT core motifs.

#### Analysis of PHO and SFMBT binding to PREs in alternative chromatin states

The adjusted average binding signals of PHO and SFMBT at selected PREs were separately computed from two replicate ChIP-chip experiments in Sg4 and BG3 cells. To derive the adjusted average binding signal a mean of all smoothed IP/INPUT values within 500 bp regions centered on PHO peak at a PRE was computed and the genomic average IP/INPUT value (background signal) was subtracted from it. The statistical significance of the difference between adjusted average binding signals in repressed and active chromatin states was estimated by Wilcoxon signed rank test as implemented in R environment for statistical computing and graphics (http://www.r-project.org/).

### Accession numbers

All ChIP-chip data generated in this study are deposited in the NCBI Gene Expression Omnibus (GEO) under accession number GSE41854.

## Supporting Information

Figure S1 The loss of PHOL or/and PHO in cultured cells has no detectable effect on the expression of genes closest to their TSS-proximal sites. A fraction of the RNAi treated cells used for replicate ChIP experiments on Figure 1D and Figure 3 was harvested for RNA isolation and the expression of neighboring genes at both sides of the Chd1 (Chd1; Bem26), kis-1 (kis; Rpp30) and dom (dom; CG30394) PHOL/PHO TSS-proximal binding sites was measured by RT-qPCR. Two independent replicate experiments (**A**, **B**) demonstrate that the loss of PHOL or PHO or both proteins has no detectable effect on the expression of tested genes in cultured cells.
(PDF)

Figure S2 The effect of RNAi knock-down on the overall amounts of nuclear PHOL and PHO. Serial dilutions of nuclear protein from mock-treated cells and cells treated with dsRNA against PHO or PHOL were transferred to PVDF membrane and probed with indicated antibodies (**A**, **C**). Coomassie stained SDS-PAGE gels (**B**, **D**) were used as loading controls. The amount of nuclear extracts (NE) loaded to each lane is indicated above each image. The weights of molecular standards (in kDa) are shown to the left.
(PDF)

Figure S3 The PHOL binding to TSS-proximal sites does not increase after the RNAi knock-down of PHO. Chromatin from cells subjected to RNAi against PHO, PHOL or a combination of the two was immunoprecipitated with antibodies against PHOL (**A**) or PHO (**B**) proteins. The binding of either protein to a selected set of TSS-proximal sites (indicated below x-axes) does not change after the RNAi knock-down of the corresponding counterpart suggesting that at these sites PHO and PHOL do not compete. The mean of two to three independent ChIP experiments and the standard deviation (error bars) are shown. In both panels, the “*w-pr*” amplicon, which spans the promoter of *white* gene, is added as a negative control.
(PDF)

Figure S4 The RNAi knock-down of PHOL does not enhance the binding of PHO to PREs. Chromatin from cells subjected to RNAi against PHO, PHOL or a combination of the two was immunoprecipitated with antibodies against PHO protein. As expected, the binding of PHO is reduced after PHO or double PHO+PHOL knockdown but it is not affected by the single knock-down of PHOL. The mean of two independent ChIP experiments and the scatter (error bars) are shown.
(PDF)

Figure S5 SFMBT knock-down does not affect the binding of PHO and PHOL to TSS-proximal sites. Chromatin from cells subjected to SFMBT or mock-RNAi was immunoprecipitated with antibodies against SFMBT, PHO and PHOL proteins. As indicated by qPCR analysis of a selected set of TSS-proximal sites the SFMBT knock-down results in its loss from the sites (A) but has no effect on the binding of PHO (B) or PHOL (C). The mean of two to three independent ChIP experiments and the standard deviation (error bars) are shown.
(PDF)

Figure S6 The effects of PSC/SU(Z)2 deletion on PRC1 components and expression of PcG target genes. Chromatin from cultured cells carrying homozygous *Su(z)2-1.b8* deletion (white bars) or control wild type cells (black bars) was immunoprecipitated with antibodies against PC (**A**) or dRING (**B**). Here and below the mean result of two independent experiments and the scatter (error bars) are shown. The loss of PSC from PREs is paralleled by the loss of PC and dRING. **C**. RT-qPCR analysis indicates that in *Su(z)2-1.b8* cells the transcription of some PcG target genes increases but generally remains low. This does not correlate with the loss of PhoRC binding to PREs. The transcription through the control intergenic region (IR) represents the genomic background.
(PDF)

Figure S7 SFMBT and SCM in Drosophila and man. As illustrated by the comparison of the SCM (**A**) and SFMBT (**B**) proteins from *Drosophila* and man the SFMBT-SCM link is likely “broken” in humans. The comparison of *Drosophila* SCM and orthologous human proteins shows that the latter lack the zinc-finger domain required for interaction with SFMBT. Also in contrast to *Drosophila* SFMBT, human proteins with four MBT domains (grey rectangles) lack either SAM (polygons) or Zn-finger (stars) domains. Human proteins are ordered (from top to bottom) reflecting the similarity of their MBT domains to those of *Drosophila* counterpart. SAM and MBT domains are color coded to indicate relationships. Note that that the SAM domains of SFMBT1 and SFMBT2 are not related to that of SFMBT.
(PDF)

Figure S8 The extent of overlapping between YY1 bound regions detected with different anti-YY1 antibodies and individual PcG proteins or active TSS in NT2-D1 cells. The extent of overlapping between YY1 bound regions detected with all three antibodies (**A**), sc281 antibodies (**B**) and sc7341 (**C**) antibodies was plotted as the weaker binding sites were progressively removed from the data sets. There is a clear trend for strong YY1 sites to reside within 700 bp of active TSS and virtually no overlap with PcG or H3K27me3. The very low level overlap between weak YY1 sites detected with sc281 (**B**) and sc7341 (**C**) antibodies and PcG/H3K27me3 is due to noise.
(PDF)

Figure S9 The regions bound by YY1 in K562 cells are near active TSS and do not overlap PcG proteins or H3K27me3. The extent of YY1 overlapping is plotted as function of YY1 binding strength. Most of the sites reside within 700 bp of active TSS and none overlap with PcG or H3K27me3.
(PDF)

Table S1 The levels of PhoRC components at different computationally defined PREs.
(XLSX)

Table S2 Correlation between PHO, PHOL and dSFMBT at computationally defined PREs.
(XLSX)

Table S3 The binding of PHO and SFMBT to PREs of active target genes is reduced compared to that when the genes are repressed.
(XLSX)

Table S4 The list of PCR primers.
(XLSX)
