# Supplementary figures and images for: Combinatorial Interactions Are Required for the Efficient Recruitment of Pho Repressive Complex (PhoRC) to Polycomb Response Elements
Source: PLoS Genet. 2014 Jul 10;10(7):e1004495. doi: 10.1371/journal.pgen.1004495 (PMC4091789; doi:10.1371/journal.pgen.1004495)

**A**

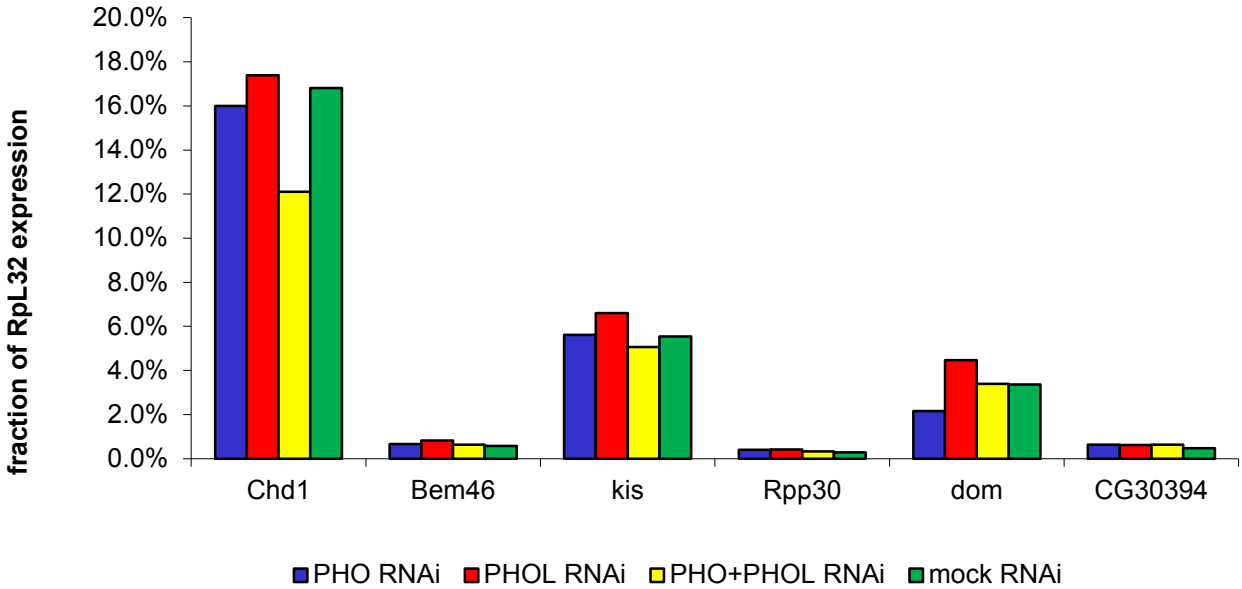

**B**

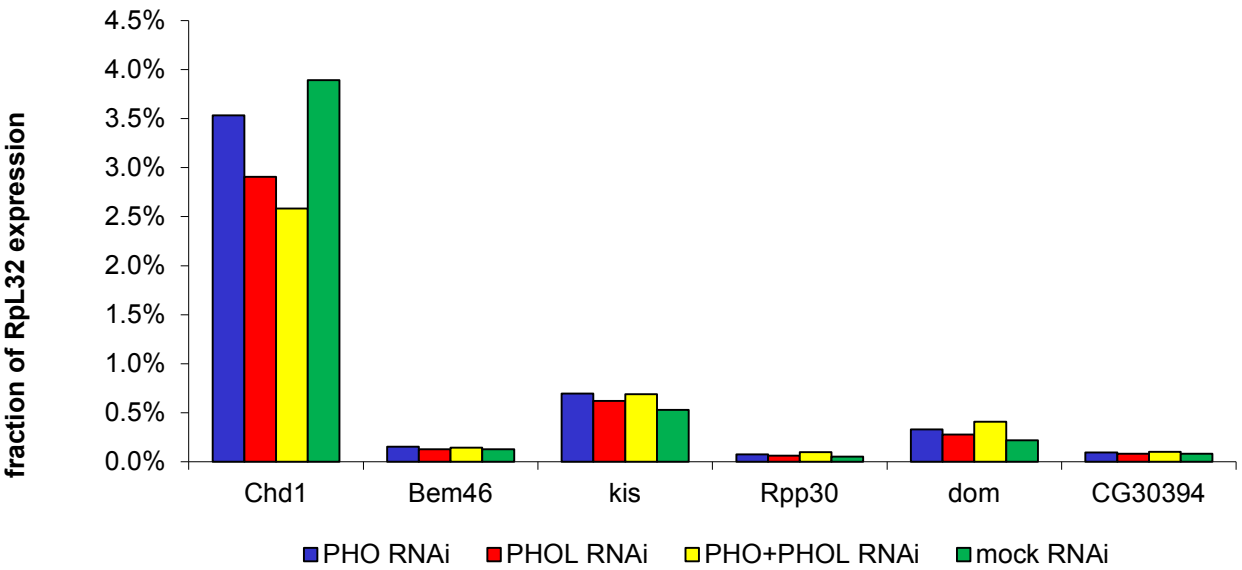

Supplement: Figure S1 — The loss of PHOL or/and PHO in cultured cells has no detectable effect on the expression of genes closest to their TSS-proximal sites. A fraction of the RNAi treated cells used for replicate ChIP experiments on Figure 1D and Figure 3 was harvested for RNA isolation and the expression of neighboring genes at both sides of the Chd1 (Chd1; Bem26), kis-1 (kis; Rpp30) and dom (dom; CG30394) PHOL/PHO TSS-proximal binding sites was measured by RT-qPCR. Two independent replicate experiments (A, B) demonstrate that the loss of PHOL or PHO or both proteins has no detectable effect on the expression of tested genes in cultured cells. (PDF) [file pgen.1004495.s001.pdf]

**A**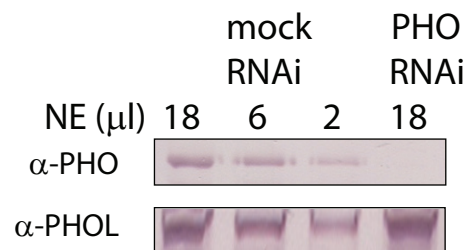**B**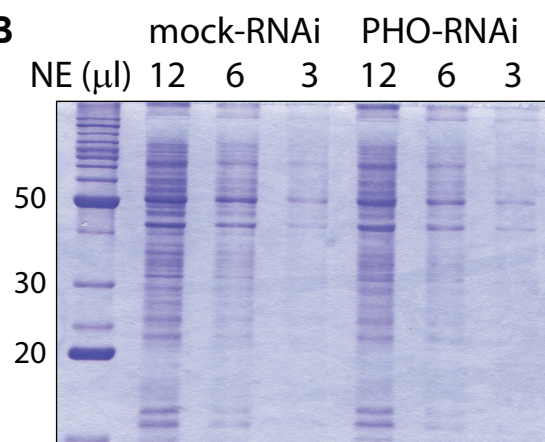**C**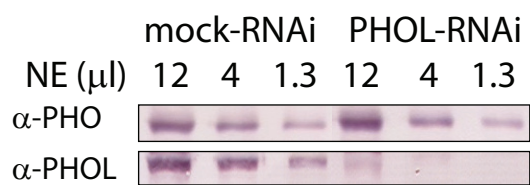**D**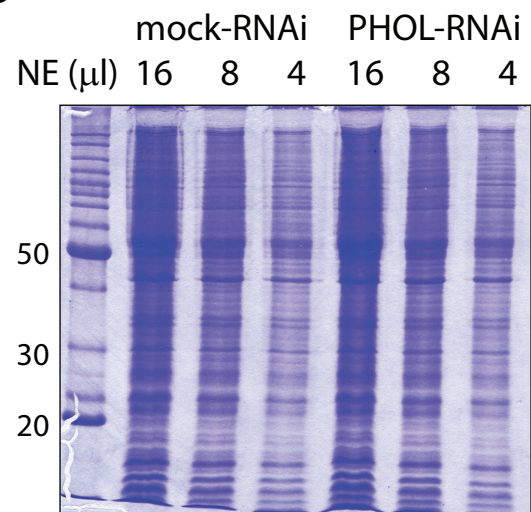

Supplement: Figure S2 — The effect of RNAi knock-down on the overall amounts of nuclear PHOL and PHO. Serial dilutions of nuclear protein from mock-treated cells and cells treated with dsRNA against PHO or PHOL were transferred to PVDF membrane and probed with indicated antibodies (A, C). Coomassie stained SDS-PAGE gels (B, D) were used as loading controls. The amount of nuclear extracts (NE) loaded to each lane is indicated above each image. The weights of molecular standards (in kDa) are shown to the left. (PDF) [file pgen.1004495.s002.pdf]

A

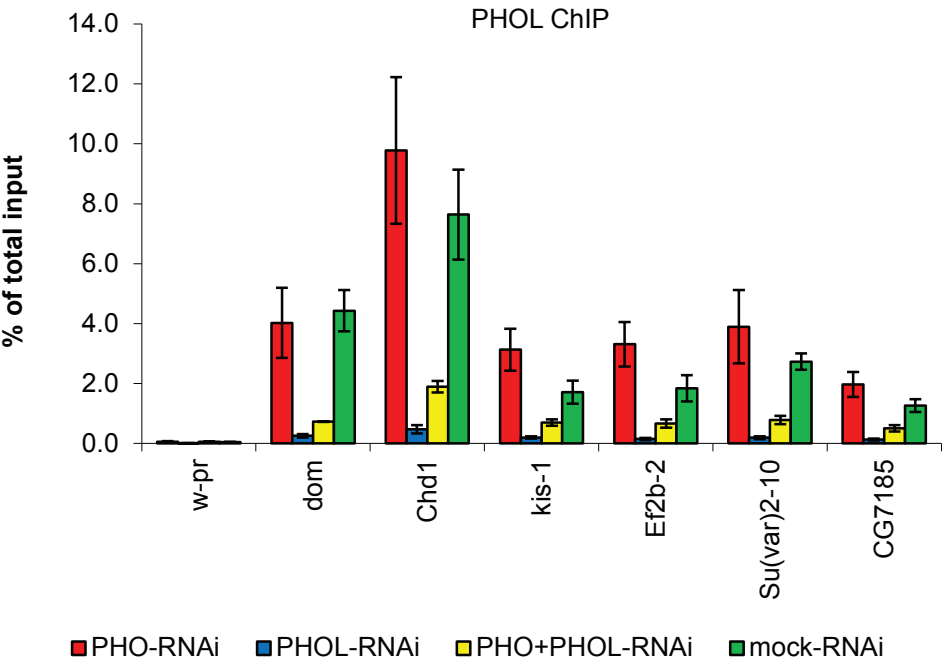

B

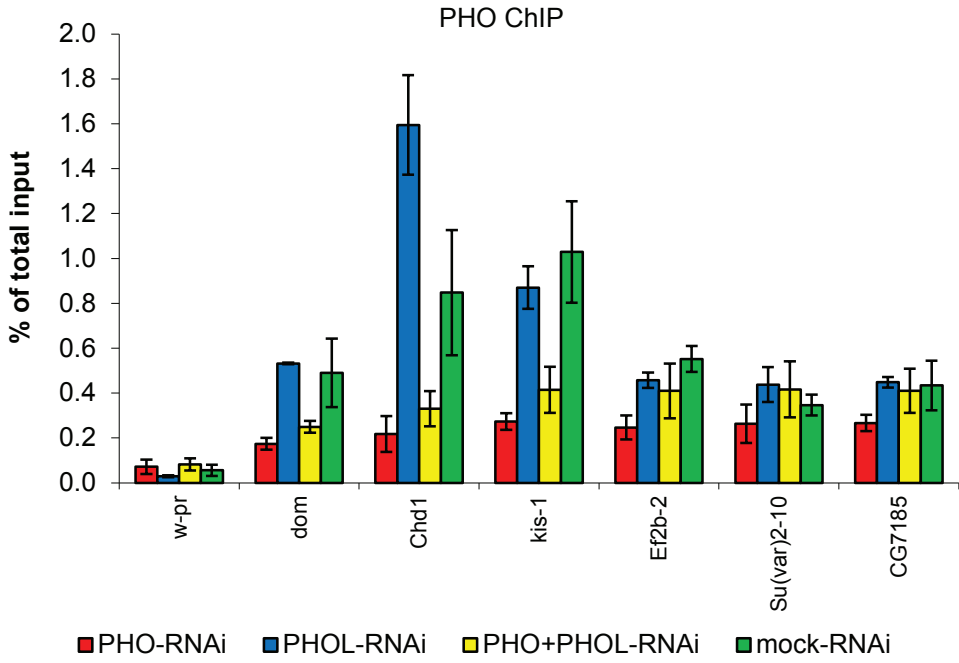

Supplement: Figure S3 — The PHOL binding to TSS-proximal sites does not increase after the RNAi knock-down of PHO. Chromatin from cells subjected to RNAi against PHO, PHOL or a combination of the two was immunoprecipitated with antibodies against PHOL (A) or PHO (B) proteins. The binding of either protein to a selected set of TSS-proximal sites (indicated below x-axes) does not change after the RNAi knock-down of the corresponding counterpart suggesting that at these sites PHO and PHOL do not compete. The mean of two to three independent ChIP experiments and the standard deviation (error bars) are shown. In both panels, the “w-pr” amplicon, which spans the promoter of white gene, is added as a negative control. (PDF) [file pgen.1004495.s003.pdf]

Kahn Figure S4

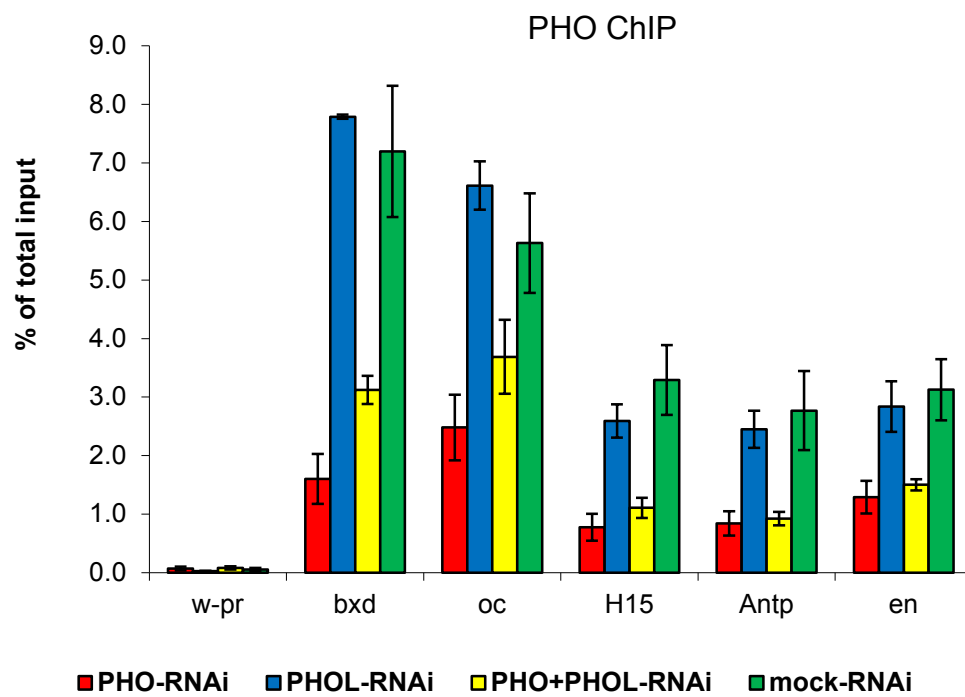

Supplement: Figure S4 — The RNAi knock-down of PHOL does not enhance the binding of PHO to PREs. Chromatin from cells subjected to RNAi against PHO, PHOL or a combination of the two was immunoprecipitated with antibodies against PHO protein. As expected, the binding of PHO is reduced after PHO or double PHO+PHOL knockdown but it is not affected by the single knock-down of PHOL. The mean of two independent ChIP experiments and the scatter (error bars) are shown. (PDF) [file pgen.1004495.s004.pdf]

**A**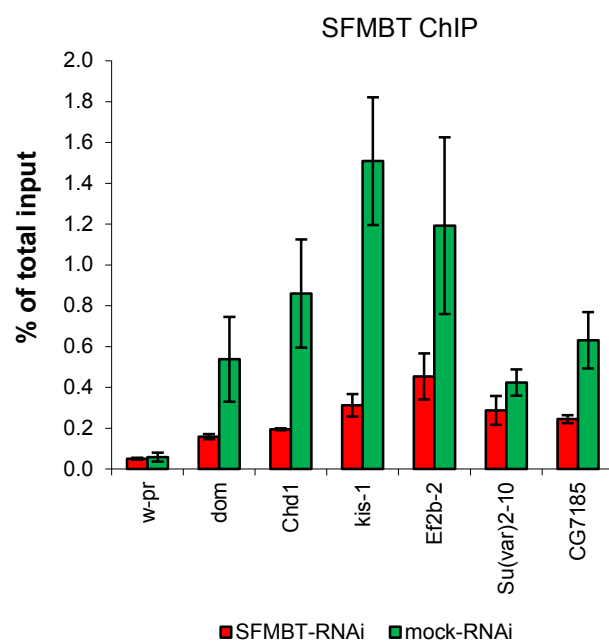**B**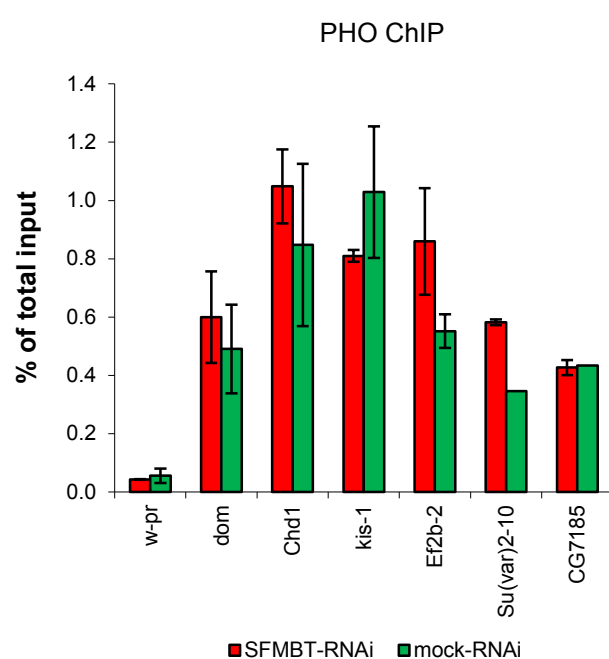**C**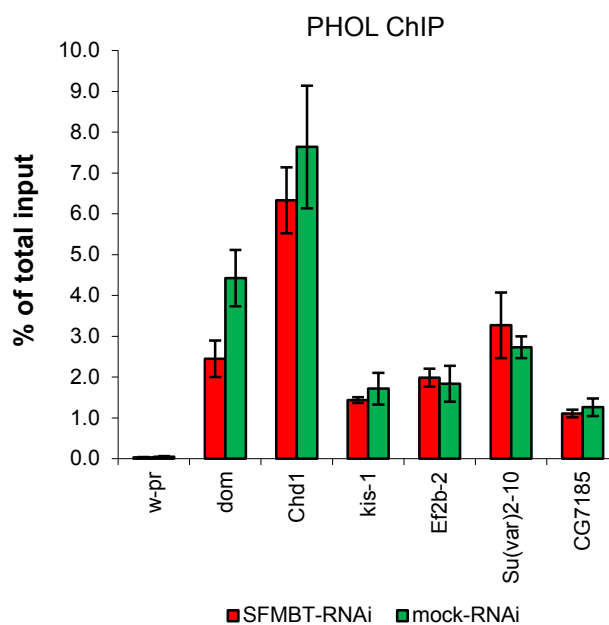

Supplement: Figure S5 — SFMBT knock-down does not affect the binding of PHO and PHOL to TSS-proximal sites. Chromatin from cells subjected to SFMBT or mock-RNAi was immunoprecipitated with antibodies against SFMBT, PHO and PHOL proteins. As indicated by qPCR analysis of a selected set of TSS-proximal sites the SFMBT knock-down results in its loss from the sites (A) but has no effect on the binding of PHO (B) or PHOL (C). The mean of two to three independent ChIP experiments and the standard deviation (error bars) are shown. (PDF) [file pgen.1004495.s005.pdf]

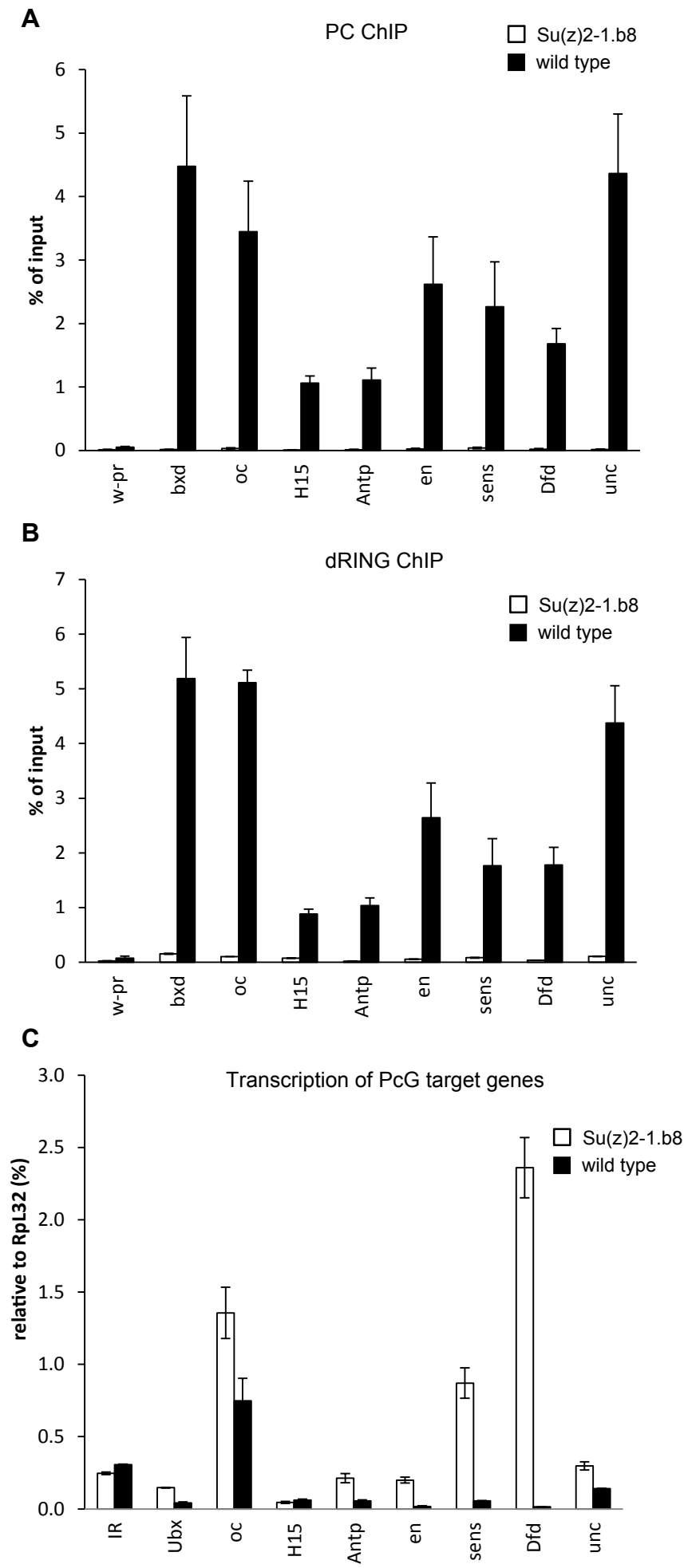

Supplement: Figure S6 — The effects of PSC/SU(Z)2 deletion on PRC1 components and expression of PcG target genes. Chromatin from cultured cells carrying homozygous Su(z)2-1.b8 deletion (white bars) or control wild type cells (black bars) was immunoprecipitated with antibodies against PC (A) or dRING (B). Here and below the mean result of two independent experiments and the scatter (error bars) are shown. The loss of PSC from PREs is paralleled by the loss of PC and dRING. C. RT-qPCR analysis indicates that in Su(z)2-1.b8 cells the transcription of some PcG target genes increases but generally remains low. This does not correlate with the loss of PhoRC binding to PREs. The transcription through the control intergenic region (IR) represents the genomic background. (PDF) [file pgen.1004495.s006.pdf]

A

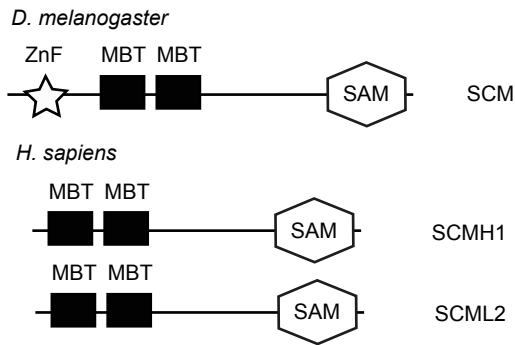

B

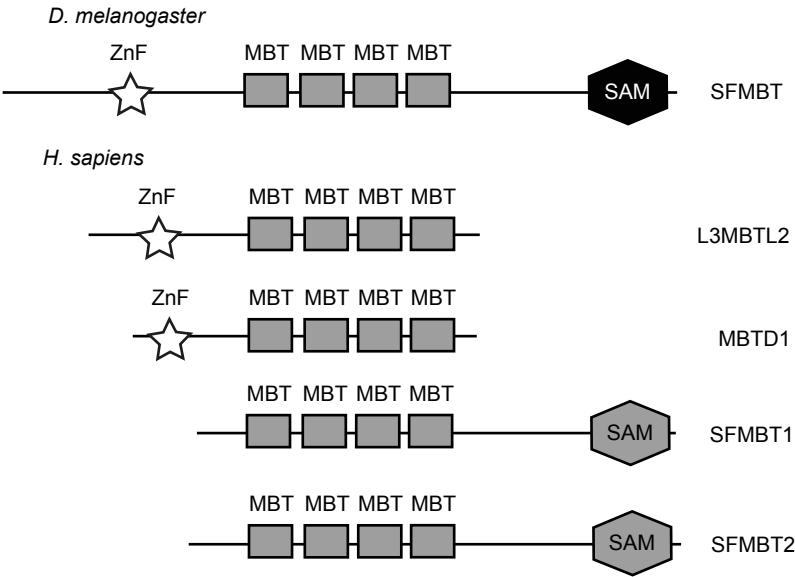

Supplement: Figure S7 — SFMBT and SCM in Drosophila and man. As illustrated by the comparison of the SCM (A) and SFMBT (B) proteins from Drosophila and man the SFMBT-SCM link is likely “broken” in humans. The comparison of Drosophila SCM and orthologous human proteins shows that the latter lack the zinc-finger domain required for interaction with SFMBT. Also in contrast to Drosophila SFMBT, human proteins with four MBT domains (grey rectangles) lack either SAM (polygons) or Zn-finger (stars) domains. Human proteins are ordered (from top to bottom) reflecting the similarity of their MBT domains to those of Drosophila counterpart. SAM and MBT domains are color coded to indicate relationships. Note that that the SAM domains of SFMBT1 and SFMBT2 are not related to that of SFMBT. (PDF) [file pgen.1004495.s007.pdf]

A

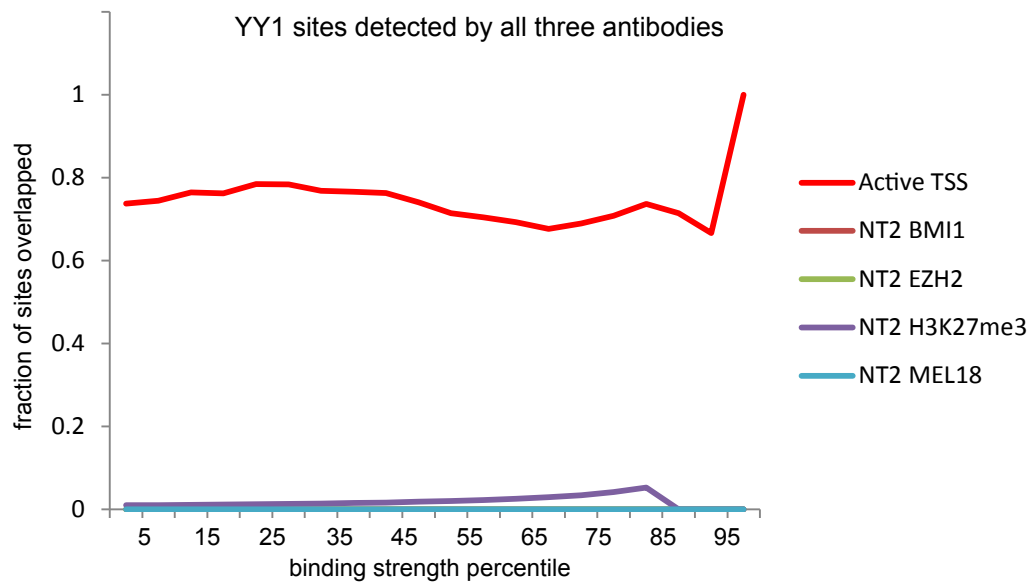

B

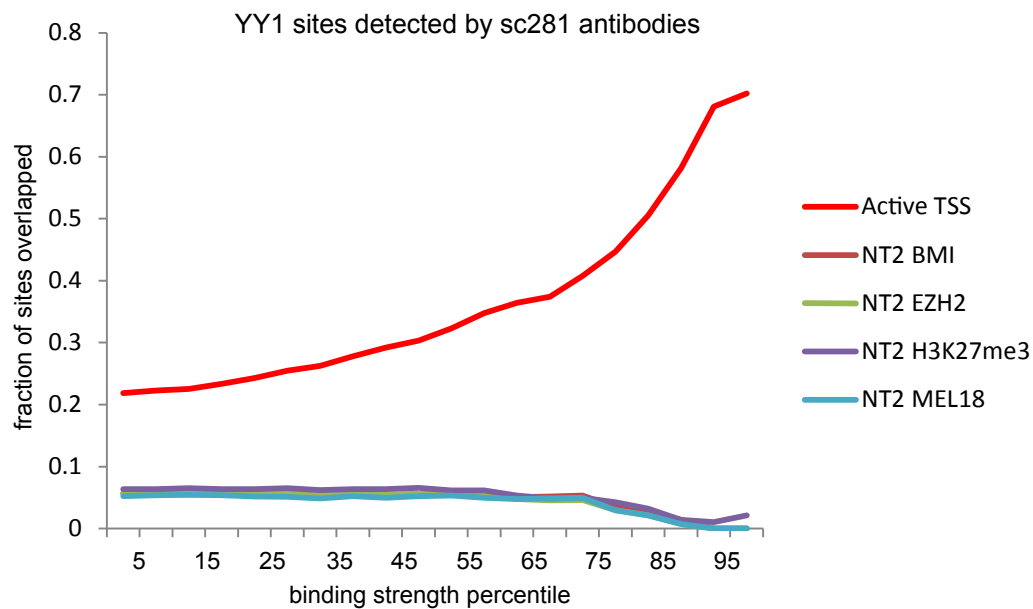

C

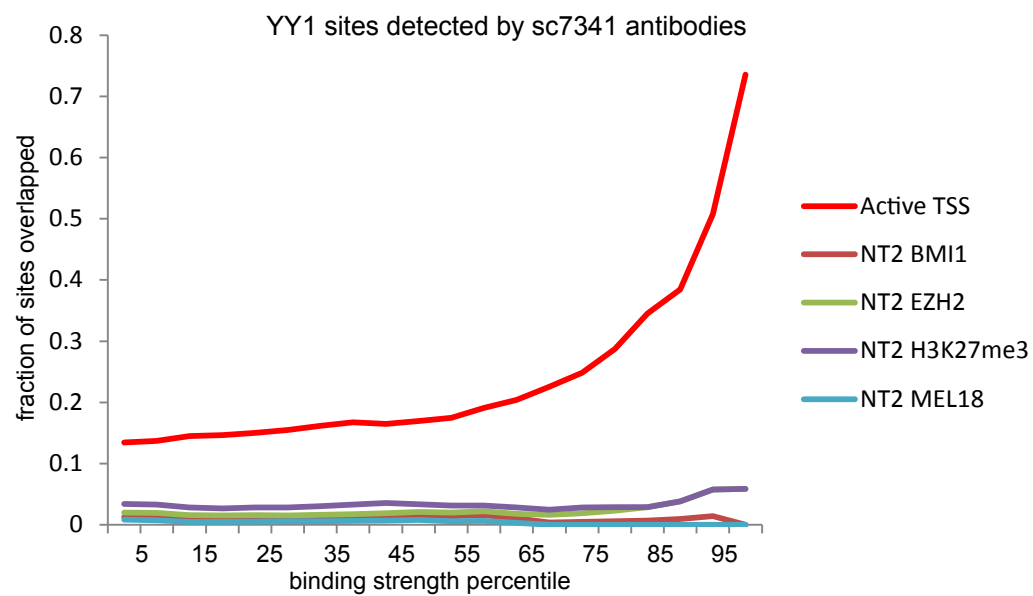

Supplement: Figure S8 — The extent of overlapping between YY1 bound regions detected with different anti-YY1 antibodies and individual PcG proteins or active TSS in NT2-D1 cells. The extent of overlapping between YY1 bound regions detected with all three antibodies (A), sc281 antibodies (B) and sc7341 (C) antibodies was plotted as the weaker binding sites were progressively removed from the data sets. There is a clear trend for strong YY1 sites to reside within 700 bp of active TSS and virtually no overlap with PcG or H3K27me3. The very low level overlap between weak YY1 sites detected with sc281 (B) and sc7341 (C) antibodies and PcG/H3K27me3 is due to noise. (PDF) [file pgen.1004495.s008.pdf]

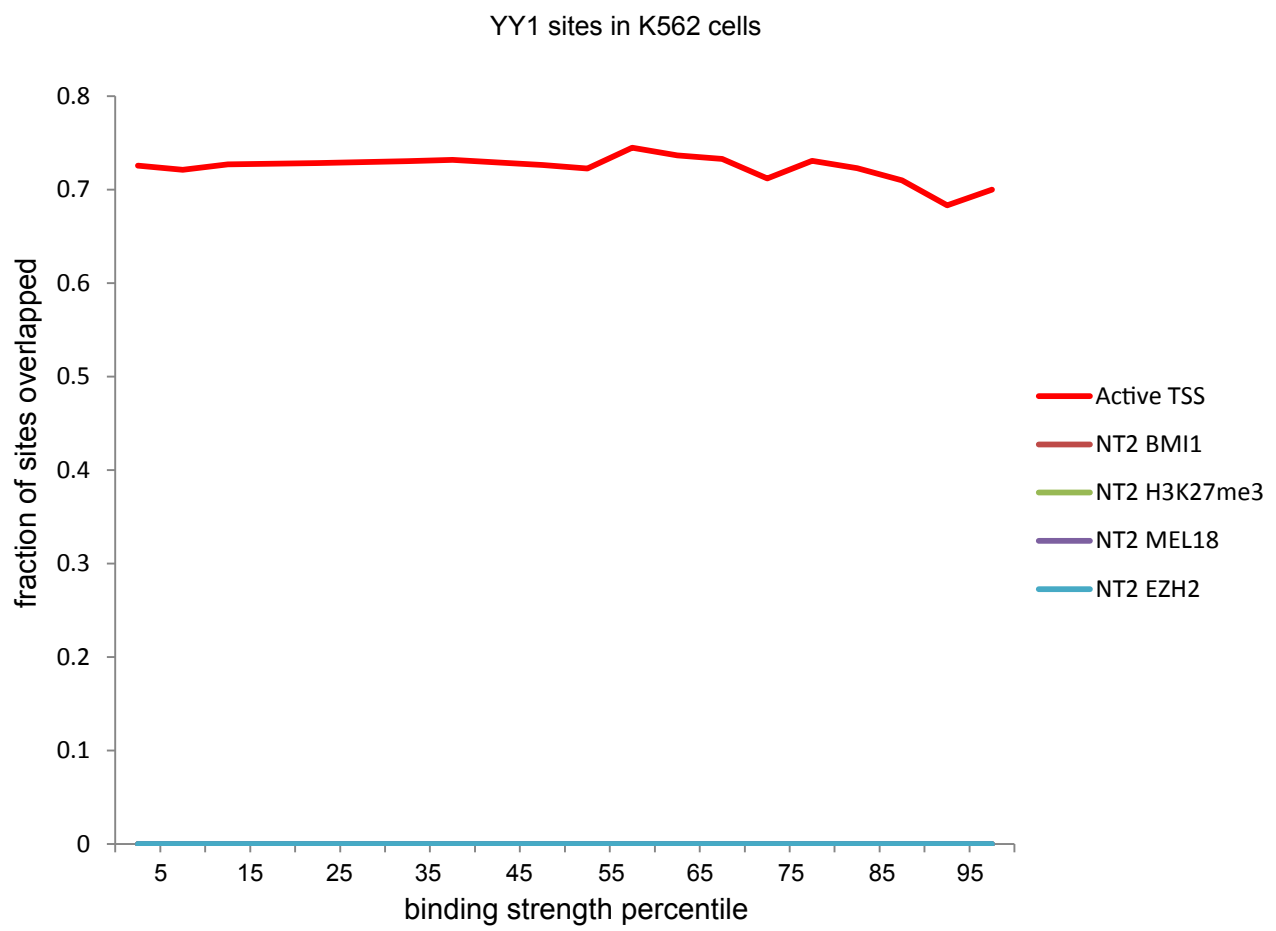

Supplement: Figure S9 — The regions bound by YY1 in K562 cells are near active TSS and do not overlap PcG proteins or H3K27me3. The extent of YY1 overlapping is plotted as function of YY1 binding strength. Most of the sites reside within 700 bp of active TSS and none overlap with PcG or H3K27me3. (PDF) [file pgen.1004495.s009.pdf]
